# Molecular Programming Design of Glyconucleic Acid Aptamer with High Stability

**DOI:** 10.1002/advs.202408168

**Published:** 2024-12-04

**Authors:** Yongqi Han, Rongjun Zhang, Hong‐Liang Bao, Mei Yang, Yuan Gao, Xiaobo Gao, Ruowen Wang, Weihong Tan, Ding‐Kun Ji

**Affiliations:** ^1^ Institute of Molecular Medicine (IMM) Renji Hospital State Key Laboratory of Oncogenes and Related Genes Shanghai Jiao Tong University School of Medicine Shanghai 200240 China; ^2^ College of Chemistry and Materials Science Shanghai Normal University Shanghai 200234 China; ^3^ Department of Anatomy and Physiology Shanghai Jiao Tong University School of Medicine Shanghai 200025 China; ^4^ Zhejiang Cancer Hospital Hangzhou Institute of Medicine (HIM) The Chinese Academy of Sciences Hangzhou Zhejiang 310022 China; ^5^ Molecular Science and Biomedicine Laboratory (MBL) State Key Laboratory of Chemo/Biosensing and Chemometrics College of Chemistry and Chemical Engineering College of Biology Aptamer Engineering Center of Hunan Province Hunan University Changsha Hunan 410082 China

**Keywords:** aptamers, DNA medicine, functional nucleic acids, molecular probes, precision medicine

## Abstract

Functional nucleic acids (FNAs), possessing specific biological functions beyond their informational roles, have gained widespread attention in disease therapeutics. However, their clinical application is severely limited by their low serum stability in complex physiological environments. In this work, a precise molecular programming strategy is explored to prepare glyconucleic acid aptamers (GNAAs) with high serum stability. Four glyconucleic acid modules compatible with commercial solid‐phase synthesis are designed and synthesized. Through precise molecular design, the accurate modification of four different carbohydrate ligands at specific sites of DNA aptamers is achieved. It is demonstrated that glycosylation modification can significantly increase DNA aptamers’ serum stability while maintaining their structures and high affinity. The stabilization effect is superior to that of currently commonly used commercial chemical modifications. Moreover, it is confirmed that this approach displays insignificant effects on the DNA aptamers’ tumor‐targeting ability and metabolism in vivo. This method offers a simple, economical, and efficient strategy for precise glycosylation modification of nucleic acids. This allows to prepare glycosyl functional nucleic acids with high serum stability, which can expand the application scope of functional nucleic acids and promote the practical transformation of functional nucleic acids.

## Graphical abstract

A precise molecular programming strategy for glycosylation of oligonucleotides is developed. This method achieves precise control over the position and types of sugar molecules on DNA aptamers. Glycosylation modification is confirmed to effectively enhance the serum stability of DNA aptamers, while preserving their unique secondary structure and high affinity for cancer cells.

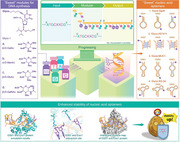

## 1 Introduction

Functional nucleic acids (FNAs)‐based therapeutics have recently garnered significant attention in disease treatment.^[^ ^1^, ^2^ ^]^ FNAs constitute a class of synthetic nucleic acids (DNA or RNA) endowed with specific biological functions beyond their informational roles.^[^ ^3^, ^4^ ^]^ Notable progress has been made in FNA drugs, exemplified by various instances that have undergone FDA‐approved clinical trials, including short interfering RNAs (siRNAs), small activating RNAs (saRNAs), microRNAs (miRNAs), and antisense oligonucleotides (ASOs).^[^ ^5^, ^6^ ^]^ Among them, aptamers, known as “chemical antibodies,” are functionally comparable to traditional antibodies, but offer several advantages, including their relatively small physical size, flexible structure, quick chemical production, versatile chemical modification, high stability, and lack of immunogenicity.^[^ ^7^ ^]^ Aptamers have broad affinity and are expected to develop more efficient molecular tools to meet future biomedical clinical needs in drug delivery, disease detection and diagnosis, and molecular recognition.^[^ ^8^ ^]^ By utilizing aptamers or their conjugations with drugs, cancer treatments based on aptamers have demonstrated exceptional therapeutic effects in preclinical studies.^[^ ^9^, ^10^, ^11^ ^]^

Although a series of successes have been achieved, exploring aptamers as therapeutics in the clinic is still challenging as they are easily degraded by nucleases, and their functions are easily affected in complex physiological environments. The first FDA‐approved aptamer‐based therapeutic agent pegaptanib (brand name Macugen) was approved in 2005 to treat age‐related macular degeneration.^[^ ^12^ ^]^ As of July 8, 2024, according to clinical trial data (Clinical Trials.gov), 59 aptamer‐related therapy reagents have entered clinical testing. Unremitting efforts have been made to avoid nucleic acid aptamer degradation and maintain their functionality in serum. Chemical modification is considered one of the most commonly used strategies to stabilize the inherent structure and function of aptamers.^[^ ^13^ ^]^ Three modification sites, including the terminal of the aptamers, the sugar ring, and the phosphodiester backbone, have been successfully achieved.^[^ ^14^ ^]^ A series of chemical approaches have been applied in clinical nucleic acid drugs.^[^ ^5^ ^]^ Additionally, many nano‐delivery technologies, such as lipid nanoparticles,^[^ ^15^, ^16^ ^]^ serum albumins,^[^ ^17^ ^]^ and nanoparticles,^[^ ^18^, ^19^ ^]^ have also been explored to enhance aptamer stability and increase their delivery both in vitro and in vivo. However, many existing methods of aptamer modification are time‐consuming and labor‐intensive. Furthermore, there is increasing evidence that some current classic modifications could affect the function of aptamers or introduce immunogenicity issues.^[^ ^20^, ^21^ ^]^ In this regard, a simple, safe, and effective method for enhancing the stability of aptamers and maintaining their functionalities is still highly desired.

Recently, Bertozzi's group reported the successful isolation of glycoRNA, which remains stable on cell membranes.^[^ ^22^ ^]^ This discovery challenges the traditional concept of natural biological macromolecules. It confirms that in addition to proteins and lipids, glycosylation represents a third major carrier and suggests that glycoRNA may serve important physiological functions on the surface of cell membranes. This finding introduces a novel concept for the development of future research directions.^[^ ^23^, ^24^ ^]^ Additionally, the modification of Tri‐GalNAc structures with siRNA oligos has resulted in significant advancements in therapeutic delivery. Currently, several GalNAc‐siRNA conjugates are being assessed in clinical trials for the treatment of various diseases.^[^ ^25^ ^]^ These findings confirm that carbohydrates can increase oligonucleotides’ systemic stability against nucleases and improve their functionality. Carbohydrate molecules could be considered one of the promising candidates in the research on long‐acting aptamer modification. Although some covalent conjugation of carbohydrates, especially GalNAc, to oligonucleotides for the delivery and targeting of potential nucleic acid therapeutics has been reported.^[^ ^26^, ^27^, ^28^, ^29^ ^]^A general strategy for preparing glyconucleic acid aptamers (GNAAs) has rarely been reported, and the impact of the conformational stability of the aptamers on their performance remains unknown.

**Figure 1:**
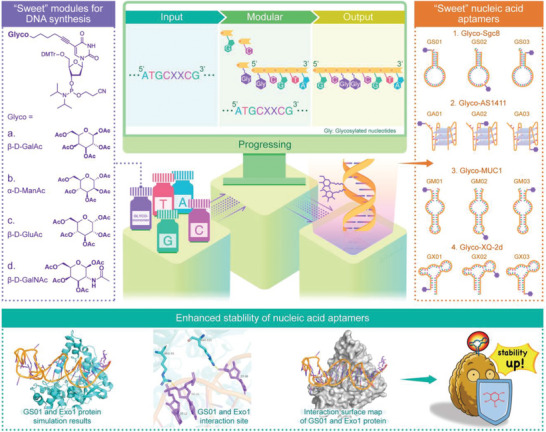
Schematic diagram of the preparation of glyconucleic acid aptamers (GNAAs) via precise and programmable DNA solid‐phase synthesis techniques.

Here, we present a molecular programming strategy to prepare glyconucleic acid aptamers (GNAAs) with enhanced stability through DNA solid‐phase synthesis technology. In this work, four glyconucleic acid modules were designed and synthesized to be suitable for commercial solid‐phase synthesis. Through precise molecular design, we have achieved the accurate modification of four different carbohydrate ligands at specific sites of DNA aptamers. A series of GNAAs with carbohydrates at the 3′ terminal, 5′ terminal, and an intermediate site were successfully prepared and comprehensively characterized. Furthermore, their stability and functions were studied both in vitro and in vivo. We demonstrate that terminal glycosylation modification can significantly increase the serum stability of DNA aptamers while maintaining their structures and high affinity (**Figure** **1**). This work provides a universal approach to prepare glycosylated FNAs with high stability, that is expected to expand the application scope of FNAs and accelerate the clinical transformation of aptamer‐based therapeutic agents.

## 2 Results and Discussion

### 2.1 Design, Synthesis, and Characterization of Glyconucleic Acid Modules and GNAAs

**Scheme 1:**
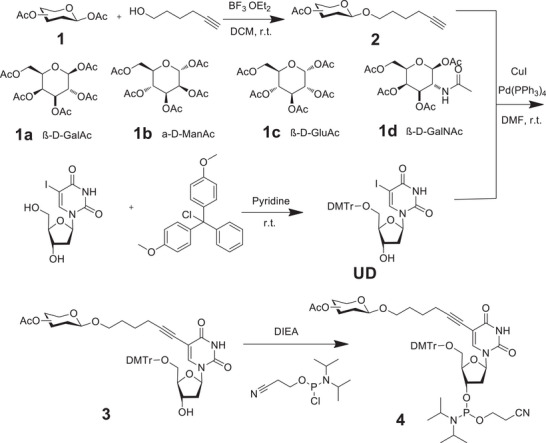
Synthesis of sugar module phosphorimide monomer. Four natural glycoligands (galactose, N­acetyl galactosamine, glucose, and mannose) were chosen as raw materials to prepare glyconucleic acid modules via a four‐step organic chemical reaction.

To achieve the precise preparation of GNAAs, four natural glycoligands (i.e., galactose [Gal], N­acetyl galactosamine [GalNAc], glucose [Glu], and mannose [Man]) were chosen as raw materials to form glyconucleic acid modules, that are suitable for solid‐phase DNA synthesis. These sugar‐modified oxyuridine phosphoramidite derivatives were synthesized through a four‐step organic chemical reaction (see **Scheme** **1**). Briefly, after obtaining fully acetylated sugars with a terminal alkyne portion of six carbons at the heterotope site through a classical Fischer glycosylation reaction, we utilized a Sonogashira cross‐coupling reaction to connect the glycoside portion to the pyridine ring of uracil using standard procedures. Subsequently, a nucleophilic substitution reaction added the phosphorite monomer to the deoxyribose site 3. Their successful synthesis was confirmed through NMR spectra of hydrogen, carbon, phosphorus, and mass spectrometry (Figures S1–S30, Supporting Information). Solid‐phase synthesis of GNAAs was performed on an automated Expedite Nucleic Acid Synthesis System from the 3′ to the 5′ end by using benzimidazolium triflate as an activator (1.0 mmol scale). The GNAAs were deprotected and cleaved from the resin with aqueous ammonia at room temperature, then purified by a Sephadex G‐25 cartridge column and reversed‐phase HPLC. The GNAAs were produced as the main product with 90% coupling efficiency. GNAAs were prepared with different modification sites, purified by HPLC, and verified by mass spectrometry (Figures S31–S37, Supporting Information).

### 2.2 The Serum Stability of GNAAs with Different Carbohydrate Modification

**Figure 2:**
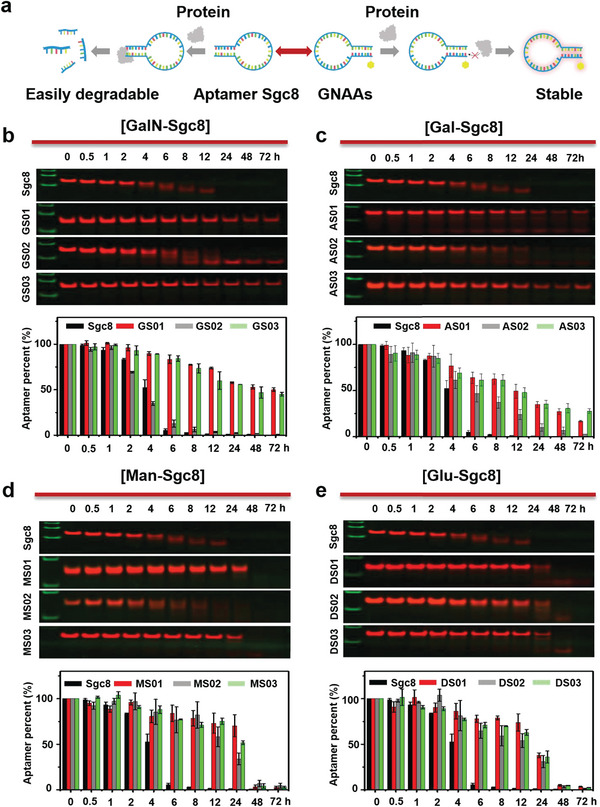
The serum stability of GNAAs with different carbohydrate modification sites and types. a) Schematic diagram of improving the serum stability of glycosylated nucleic acids (GNAAs). Serum stability of b) GalN‐Sgc8, c) Gal‐Sgc8, d) Man‐Sgc8, and e) Glu‐Sgc8 after incubation with 10% FBS for 72 h, analyzed by PAGE and fluorescence quantitative analysis.

To demonstrate the effect of glycosylation on aptamer's stability, we selected the DNA aptamer Sgc8 as the first research object (**Figure** **2**a). It was previously screened by our group that can bind to PTK7 protein overexpressed on the membrane of colorectal cancer HCT116 cells.^[^ ^30^ ^]^ Sgc8 aptamer was precisely modified with N‐Acetylgalactosamine on the 5′ terminal, 3′ terminal, and an intermediate site, respectively. The serum stability test of aptamers was carried out in 10% FBS for 72 h by PAGE and fluorescence quantitative analysis. As depicted in Figure 2b, ≈50% of the unmodified Sgc8 was degraded rapidly within 4 h. After 6 h, ≈94% of Sgc8 was degraded. The glycosylated Sgc8 (GS02), modified in the intermediate position, showed a similar low stability as Sgc8. The modification site of GS02 is at the 12th position of the Sgc8 aptamer (Table S1, Supporting Information). Interestingly, the GalN‐Sgc8s modified at the 5′ (GS01) and 3′ positions (GS03) showed a highly extended stability under the same test conditions. After 6 h, ≈85% of GS01 and GS03 were kept stable in the test solution, suggesting a 14‐fold increase of stability higher than Sgc8. Even 72 h later, 50% of GS01 and GS03 can be detected. The 3′ inverted dT modification is one of the most widely used commercial chemical modification strategies to enhance the serum stability of oligonucleotides. Over twenty nucleic acid aptamer drugs with 3′ inverted dT have been approved for clinical use or reported in phase II/III studies.^[^ ^7^, ^30^ ^]^ Thus, we compared the serum stability of our 3′ glycosylation‐modified Sgc8 (GS03) with the 3′ inverted dT‐modified Sgc8 (IS01). We found that GS03 displayed higher serum stability than IS01 (Figure S38, Supporting Information). After 24 h, over 90% of ISO1 was degraded, while ≈60% of GS03 remained intact. It is noteworthy that ≈50% of GS01 remained stable after 72 h. These results indicated that terminal carbohydrate modification can significantly increase the stability of DNA aptamers.

### 2.3 The Serum Stability of GNAAs with Different Types of Carbohydrates

To verify if carbohydrate types could affect the stability of aptamers, we next conducted the serum stability tests on mannose‐modified Sgc8 (Man‐Sgc8s), glucose‐modified Sgc8 (Glu‐Sgc8s), and galactose‐modified Sgc8 (Gal‐Sgc8s). According to the PAGE results shown in Figure 2c–e, similar stability to GalN‐Sgc8s was observed. Glyco‐Sgc8s modified at the 5′ (MS01, DS01, AS01) and 3′ (MS03, DS03, AS03) sites showed significantly improved stability in FBS. After 12 h, over 50% of these GNAAs were detected in the solution, while <1% of Sgc8 could be detected. It is noteworthy that Man‐Sgc8s and Glu‐Sgc8s exhibited lower stability than Gal‐Sgc8s and GalN‐Sgc8s, with GalN‐Sgc8s showing the highest serum stability. Additionally, three glycol‐Sgc8s (MS02, DS02, AS02) modified at intermediate sites also increased the stability of Sgc8, although not as strongly as the terminal modifications. All of the above data indicated that the stability of the DNA aptamer Sgc8 could be greatly improved by terminal glycosylation modification. Moreover, the serum stability of DNA aptamers could be precisely controlled by adjusting the position and type of the carbohydrate moiety.

### 2.4 The Serum Stability of Various GNAAs

**Figure 3:**
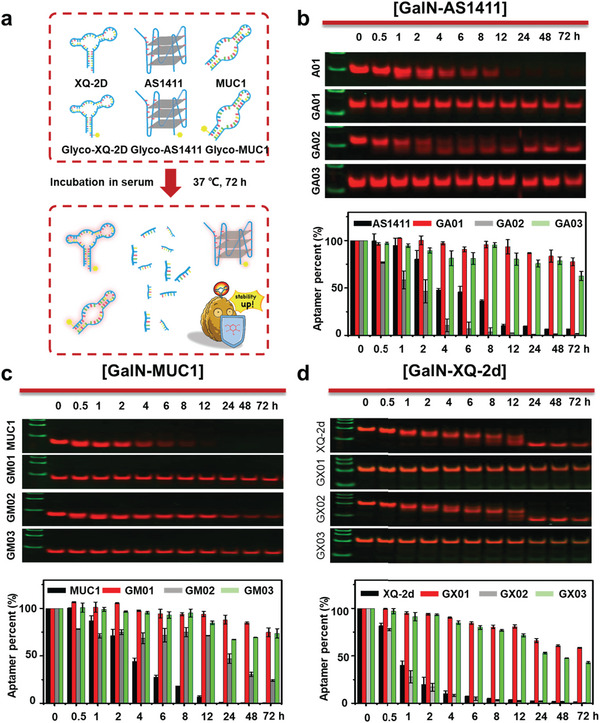
The serum stability of other GNAAs with different carbohydrate modification sites. a) Schematic diagram of improving the stability of other GNAAs. Serum stability of b) GalN‐AS1411, c) GalN‐MUC1, and d) GalN‐XQ‐2d after incubation with 10% FBS for 72 h, analyzed by PAGE and fluorescence quantitative analysis.

We next ask whether glycosylation modification could be used to increase the stability of other DNA aptamers. Three well‐known DNA aptamers with different bases and varying tertiary structures were engineered to be GNAAs (**Figure** **3**a). They are AS1411 which has 26 bases and can form a special G‐quadruplex structure,^[^ ^30^ ^]^ MUC1 with 25 bases,^[^ ^32^ ^]^ and XQ‐2d with 56 bases.^[^ ^33^ ^]^ All of them are functional oligonucleotides that are widely used in biomedical applications.^[^ ^8^ ^]^ Three aptamers were precisely modified with a GalNAc base at the intermediate site and 3′ and 5′ terminal sites. The serum stability test of the aptamers was carried out in 10% FBS for 72 h by PAGE and fluorescence quantitative analysis. As shown in Figure 3b–d, unmodified AS1411, MUC1, and XQ‐2d degrade rapidly in serum. After 12 h, over 90% of unmodified aptamers were degraded. Intermediate‐modified AS1411 (GA02) and XQ‐2d (GX02) displayed similar stability to their unmodified aptamers, respectively. Interestingly, the intermediate‐modified MUC1 (GM02) displayed enhanced stability. After 12 h, ≈75% of GM02 remained stable in serum. As we observed in the glycosylated‐Sgc8 test, all of the 3′ and 5′ modified GalN‐aptamers showed extremely high stability in serum. Over 75% of these glycosylated aptamers remained stable after 12 h of incubation. Even after 72 h, ≈50% of them remained stable in serum. These results are consistent with the stability findings of GalN‐Sgc8. All these data suggested that precise glycosylation could be a general strategy to increase the stability of DNA aptamers.

### 2.5 The Mechanism of Glycosylation Enhancing Aptamer Stability

**Figure 4:**
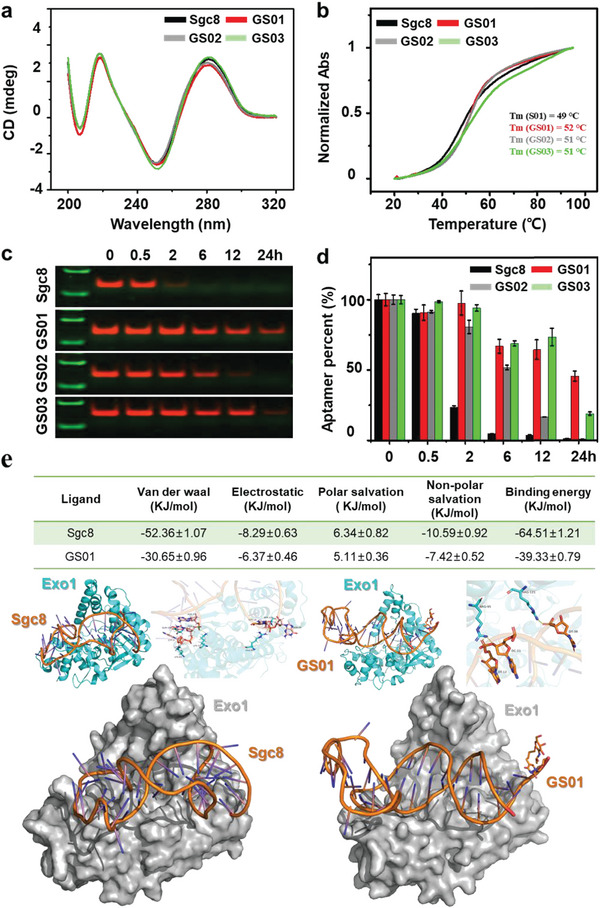
Mechanism study of glycosylation enhances the stability of GNAAs. a) Circular Dichroism of GalN‐Sgc8s. b) Tm test of GalN‐Sgc8s. c) Stability of GalN‐Sgc8s cultured by Exo1. d) Quantitative fluorescence statistics of GalN‐Sgc8s. e) Molecular simulation data and structure of Sgc8, GS01 and Exo1.

Encouraged by these results, we next attempted to clarify how glycosylation enhances the stability of aptamers using a series of techniques. Taking GalN‐Sgc8 as the representative, we first explored the GNAA's structure using Circular Dichroism (CD) (in **Figure** **4**a). The CD experiment confirmed that GalN‐Sgc8 and Sgc8 formed similar geometrical structures in an aqueous solution. This suggests that glycosylation does not affect the secondary structure of the DNA aptamer in solution. We further measured their Tm using the UV–vis spectrum. The results shown in Figure 4b indicated that glycosylation caused an insignificant effect on the Tm value of DNA aptamers. These results suggested that carbohydrate modification does not affect the structure of aptamers. Considering that nucleases are the main force in degrading nucleic acids, we hypothesized that glycosylation could hinder the interaction between nucleases and DNA aptamers. To clarify this hypothesis, GalN‐Sgc8s were incubated with 0.05 U µL^−1^ of exonuclease and endonuclease at 37 °C for various durations and then analyzed using denaturing 10% PAGE. As shown in Figure 4c,d, all of the GalN‐Sgc8s (GS01, GS02, GS03) exhibited clear resistance to exonuclease compared to unmodified Sgc8. Notably, GS01, modified with 5′ terminal glycosylation, showed the highest resistance to exonuclease. These data indicated that glycosylation can resist exonuclease cleavage, thereby increasing the stability of aptamers.

A molecular simulation was carried out to reveal the mechanism of GNAAs and Exonuclease 1 (Exo1) interaction. We build two systems, one system with modified residue on the 5′ end of aptamer (GS01), and the other system without modified residues. The GROMACS 5.0 was applied to perform the molecular dynamics simulations, where an extended simple point charge model of water (SPC/E model of water) was integrated. The crystal structure of the Exo1 protein was extracted from the protein data bank as shown in Figure 4. A total of 6 residues of Exo1 protein participate in the formation with 6 nucleic acid bases of Sgc8 (Figure 4e; Table S2, Supporting Information). At the same time, only 3 residues of Exo1 protein can interact with 3 nucleic acid bases of GS01 (Figure 4e; Table S3, Supporting Information). This indicated a weak interaction between the GS01 and Exo1. We further calculated the binding energy between Sgc8 and Exo1 using the Molecular Mechanics Poisson Boltzmann Surface Area (MM‐PBSA) approach in GROMACS software. As shown in Figure 4e, the ligand Sgc8 exhibited a Van der Waals energy of −52.36 ± 1.07 KJ mol^−1^ and an electrostatic energy of −8.29 ± 0.63 KJ mol^−1^. The polar and non‐polar solvation energies were calculated to be 6.34 ± 0.82 KJ mol^−1^ and −10.59 ± 0.92 KJ mol^−1^, respectively, leading to a total binding energy of −64.51 ± 1.21 KJ mol^−1^. In comparison, GS01 showed a slightly less favorable Van der Waals energy of −30.65 ± 0.96 KJ mol^−1^ and an electrostatic energy of −6.37 ± 0.46 KJ mol^−1^. The polar solvation energy was slightly higher at 5.11 ± 0.36 KJ mol^−1^, whereas the non‐polar solvation energy was −7.42 ± 0.52 KJ mol^−1^, culminating in a total binding energy of −39.33 ± 0.79 KJ mol^−1^. Overall, GS01 demonstrates weaker interaction with the Exo1, as reflected by a less negative binding energy. This suggests that GS01 has a lower binding affinity with Exo1 compared to Sgc8, making it resistant to degradation by exonucleases.

To further confirm the reliability of our simulation results, we conducted isothermal titration calorimetry (ITC) experiments.^[^ ^34^, ^35^ ^]^ We titrated 10 µm of the nucleic acid against 1 µm of the exonuclease protein at 37 °C. As shown in Figure S39 and Table S4 (Supporting Information), the enthalpy change (ΔH) for the interaction between S01 and Exo1 was 24.24 kJ mol^−1^, the Gibbs free energy (ΔG) was −53.44 kJ mol^−1^, and the entropy change (ΔS) was 250.5 J  (mol∙ K)^−1^. For GS01 interacting with Exo1, ΔH was −48.08 kJ mol^−1^, ΔG was −18.88 kJ mol^−1^, and ΔS was −94.16 J  (mol∙ K)^−1^. These data indicate that GS01 has a lower binding affinity for Exo1 compared to S01, which allows it to resist exonuclease degradation. This finding is consistent with our molecular simulations.

We further conducted ^1^H NMR measurements of the aptamers and identified the imine peaks of Sgc8 and GS01 at ≈12–14 ppm. The peaks of Sgc8 are consistent with the previous report.^[^ ^36^ ^]^ Probing the 10 Watson–Crick base pairs shaped the secondary structure of Sgc8, which contains three paired regions. In contrast, we found a decreased intensity of G12, G9, G35, and G36, suggesting that glycosylation modification could impact the stem‐loop junction of the aptamer (Figure S40, Supporting Information). Next, we examined whether GS01 could maintain its structure after being incubated with Exo1. We acquired ^1^H NMR spectra for Sgc8 and GS01 with the addition of Exo1 at different time points (Figure S41, Supporting Information). The imino proton signals of GS01 remained intact during 24h. In contrast, the imino proton signals of Sgc8 changed rapidly, with many signals disappearing as time increased. These data demonstrated that GS01 is resistant to exonuclease cleavage and that sugar modification plays a key role in stabilizing the stem‐loop structure.

### 2.6 Binding Ability of GNAAs to Cancer Cells

**Figure 5:**
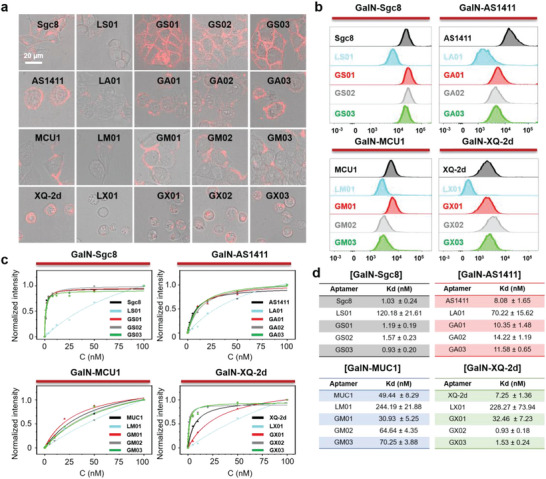
Binding ability of GNAAs. a) Confocal microscopy fluorescence images of the binding of different Cy5‐labeled GNAAs. The Sgc8 against HCT116 cells. The AS1411 against MCF‐7 cells. The MUC1 against MCF‐7 cells. The XQ‐2d pair against CCRF‐CEM cells. b) Flow cytometry‐based determination of binding affinities of Cy5‐labeled GNAAs and their parent aptamers. c) Curve fitting of KD values of different GNAAs. d) KD values of GNAAs binding to cancer cells.

We next ask whether the glycosylation could affect the biological functions of aptamer. The binding ability of GNAAs was evaluated in their related positive cancer cells. GalNAc‐modified aptamers (GalN‐Sgc8, GalN‐AS1411, GalN‐MUC1, and GalN‐XQ‐2d) with different carbohydrate modification sites were incubated with their corresponding cancer cells in binding buffer at 4 °C for 30 min, and then observed using a fluorescence confocal microscope. As shown in **Figure** **5**a, all of the unmodified aptamers and GalNAc‐modified aptamers can bind to their targeted cancer cells, respectively, while the control library chains hardly bind to cancer cells. Flow cytometry also indicated the effective biding ability of GNAAs as shown in Figure 5b. To quantitatively analyze their binding capabilities, we then measured the equilibrium dissociation constants of these aptamers in vitro using flow cytometry analysis. As shown in Figure 5c, all of the GNAAs (GalN‐Sgc8, GalN‐AS1411, GalN‐MUC1, and GalN‐XQ‐2d) displayed a high affinity at the nmol level to their corresponding cancer cells. Glycosylation did not affect the cell binding ability of Sgc8 and AS1411. Notedly, GalN‐XQ‐2d yielded a higher affinity than unmodified XQ‐2d. GX02 displayed a higher binding affinity at 0.93 ± 0.18 nm, which was ≈7 folds higher than that of XQ‐2d (Kd = 7.25 ± 1.36 nm). GX03 also displayed an improved binding affinity of Kd = 1.53 ± 0.24 nm. All data indicated that GNAA has a high affinity to cancer cells. These results suggest that precise site modification of carbohydrates can keep their high binding ability to cancer cells (Figure 5d).

### 2.7 Cell Uptake and Intracellular Trafficking Profile of GNAAs

**Figure 6:**
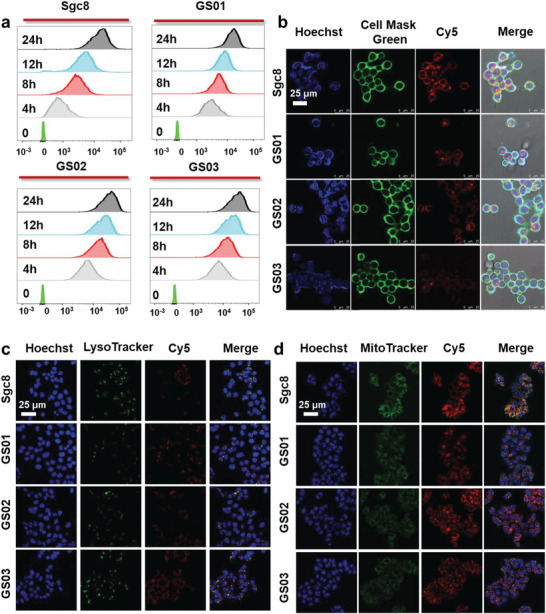
Cellular uptake behavior of the GNAAs. a) Flow cytometric assay of HCT116 cells treated with 250 nm Cy5‐labeled Sgc8 and Glyco‐Sgc8 for 0, 4, 8, 12, 24 h. b) Confocal microscopy fluorescence images of HCT116 cells incubated with 250 nm Cy5‐labeled Sgc8 or Glyco‐Sgc8 in culture medium (10% FBS) at 37 °C for 2 h. c) Confocal microscopy fluorescence images of HCT 116 cells stained with Cy5‐labeled Sgc8 or Glyco‐Sgc8 and LysoTracker Green (Ex: 488 nm, Em: 505–525 nm). d) Confocal microscopy fluorescence images of HCT 116 cells stained with Cy5‐labeled Sgc8 or Glyco‐Sgc8 and MitoTracker Green (Ex: 488 nm, Em: 505–525 nm).

We next evaluated the cell uptake and intracellular trafficking profile of GNAAs (GalN‐Sgc8, as representative aptamers) by CLSM and flow cytometry. Initially, Cy5‐labeled GalN‐Sgc8 (250 nm) were incubated with HCT116 cells at 37 °C for varying durations (0, 4, 8, 12, 24 h). Their internalization was then observed using flow cytometry. **Figure** **6**a illustrates that the intracellular fluorescence intensity progressively increased over time, indicating that internalization was time‐dependent and unaffected by glycosylation. To further visualize the internalization results, Cy5‐labeled GalN‐Sgc8 aptamers (250 nm) were incubated with HCT116 cells for 2 h to enable fluorescence confocal microscopy imaging (Figure 6b). The results also demonstrated that GalN‐Sgc8 aptamers could be successfully internalized, further suggesting glycosylation does not affect the endocytosis of aptamers. The intracellular trafficking profile of GalN‐Sgc8 was subsequently evaluated by CLSM. HCT116 cells were incubated with Cy5‐labeled GalN‐Sgc8 and Sgc8 for 2 h and co‐stained with endo/lysosome selective marker LysoTracker Green. As shown in Figure 6c, the red fluorescence from aptamers colocalizes well with the green fluorescence from LysoTracker, and Pearson's correlation is 41% for GS01, 44% for GS02, 38% for GS03, and 42% for Sgc8. At the same time, similar localization was also observed in mitochondria, as shown in Figure 6d. These results suggest that our glycosylation approach could not affect the cellular endocytosis of aptamers. Subsequently, we assessed the physiological toxicity of GalN‐Sgc8 using a CCK‐8 kit and observed no toxicity, confirming the safety of glycosylation (Figure S42, Supporting Information).

### 2.8 In Vivo Metabolism and Tumor Targeting of GNAAs

**Figure 7:**
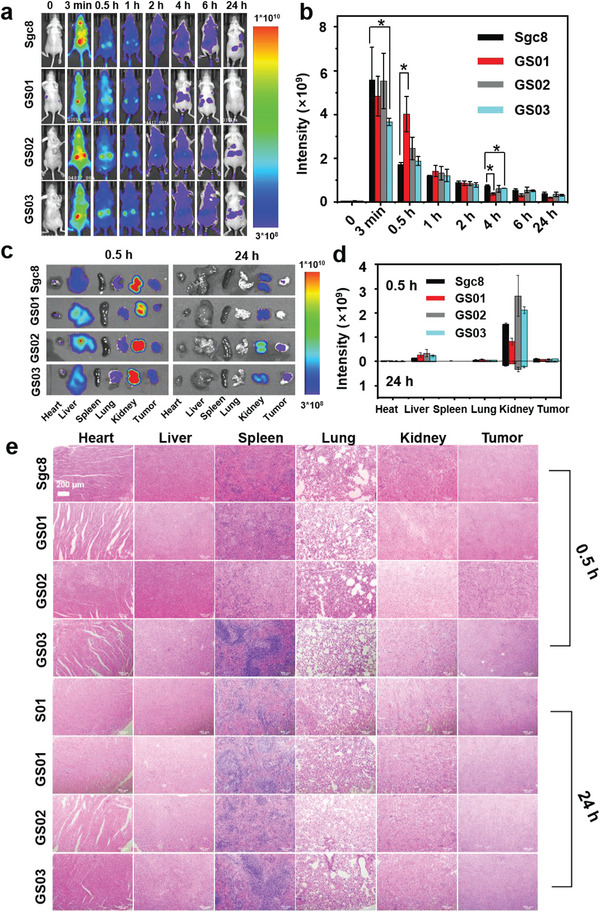
Targeting ability, distribution, and metabolism of GNAAs in vivo. a) Fluorescence distribution in vivo imaging of mice. b) Tumor site fluorescence intensity statistics. c) Organ fluorescence imaging. d) Organ fluorescence intensity statistics. e) H&E staining images of major organs of mice at different times. Data are the mean ± SD of five independent experiments (One‐way ANOVA analysis: ^*^ *p* < 0.05).

Finally, we asked how precise glycosylation might affect aptamers distribution and metabolism in vivo. Tumor xenografts were established by subcutaneously injecting HCT 116 cells (5 × 10^6^) into the lateral flanks of nude mice. When the volume of the xenograft tumors reached 5 mm × 5 mm × 5 mm, Cy5‐labeled GalN‐Sgc8, and Cy5‐labeled Sgc8 were injected at the dose of 35 µm (200 µL) per mouse via intravenous injection. Fluorescence imaging was performed at 3 min, 0.5, 1, 2, 4, 6, and 24 h, and quantitative fluorescence statistics were recorded (**Figure** **7**a,b). As shown in Figure 7a, the FL signals were full of whole mice which indicated their good dispersion. GalN‐Sgc8 rapidly went through systemic circulation and accumulated in the liver, kidney, and tumor sites. The fluorescence intensity at the tumor site rapidly enriched for 3 min and then slowly disappeared during 24 h. There were no significant differences compared to Sgc8, indicating GalN‐Sgc8 maintained their tumor targeting ability in mice. Further, ex vivo fluorescence imaging and quantitative statistics of main organs and tumors were performed at 0.5 and 24 h (Figure 7c,d). The fluorescence in the kidney gradually diminished until it was undetectable. It indicated that renal metabolism was one of the main metabolic pathways of GalN‐Sgc8. In addition, histological analyses were performed, as shown in Figure 7e. Hematoxylin and eosin (H&E) staining showed no significant cell changes in the tumor tissue after injection of different GalN‐Sgc8. In addition, no significant damage was observed in the heart, liver, spleen, lung, and kidney tissues of all GalN‐Sgc8, indicating good biosecurity of GNAAs in mice.

## 3 Conclusion

In the summary, we have provided a general method for precise glycosylation of oligonucleotides through DNA solid‐phase synthesis technology. For the first time, our approach achieves precise control of the position and types of sugar molecules on DNA aptamers. Furthermore, we demonstrated that glycosylation modification at both the 3′ and 5′ end of DNA aptamers can effectively enhance their serum stability, while preserving their unique secondary structure and high affinity to cancer cells. Moreover, we confirmed that our approach displays insignificant effects on DNA aptamers in vivo, including toxicity and metabolism. We provide a simple, economical, and efficient strategy for the molecular programming design of glyconucleic acid aptamers with enhanced stability, which could expand the application scope of aptamers. Furthermore, our proposed strategy holds great promise to be applied to other oligonucleotide therapeutics and promote the practical transformation of functional nucleic acids.

## 4 Experimental Section

### Materials

(2S,3R,4R,5R,6R)‐3‐Acetamido‐6‐(acetoxymethyl)tetrahydro‐2H‐Pyran‐2,4,5‐triyl triacetate (98%), (2R,3S,4S,5R,6R)‐6‐(Acetoxymethyl)tetrahydro‐2H‐pyran‐2,3,4,5‐tetrayl tetraacetate (98%), (2S,3R,4S,5R,6R)‐6‐(Acetoxymethyl)tetrahydro‐2H‐pyran‐2,3,4,5‐tetrayl tetraacetate (97%), 5‐Hexyn‐1‐ol (95%), 4,4′‐Dimethoxytrityl chloride (98%), 1‐((2R,4S,5R)‐4‐Hydroxy‐5‐(hydroxymethyl)tetrahydrofuran‐2‐yl)‐5‐iodopyrimidine‐2,4(1H,3H)‐dione (98%), Tetrakis (triphenylphosphine)palladium (98%) were purchased from Bide Pharmatech Ltd. (Shanghai, China). Anhydrous dichloromethane, anhydrous N, N‐dimethyl formamide (DMF), anhydrous pyridine, Triethylamine (TEA), N, N‐Diisopropylethylamine (DIEA), Sodium sulfate, 2‐Cyanoethyl N, N‐diisopropylchlorophosphoramidite (97%), and silica gel were from Adamas, (Shanghai, China). Petroleum ether, ethyl acetate, dichloromethane, and methanol were from YONGHUA CHEMICAL CO. LTD, (Jiangsu, China). Hoechst 33 342 Staining Solution for Live Cells, TEMED was purchased from Beyotime Biotechnology. 5× TBE Buffer, Premixed Powder of 1× TBE buffer, 30% Acryl/Bis solution (29:1) were from Sangon Biotech Co., Ltd. (Shanghai, China).

### Instrumentations—*Nuclear Magnetic Resonance (NMR)*

Proton nuclear magnetic resonance (^1^H NMR), carbon nuclear magnetic resonance (^13^C NMR), and phosphorus nuclear magnetic resonance (^31^P NMR) spectra were recorded on a Bruker AVANCEIII 400 MHz spectrometer using deuterated chloroform or dimethyl sulfoxide‐*d6* as solvent. The nuclear magnetic resonance (NMR) data of the aptamer were recorded on a Bruker AVANCEIII 700 MHz spectrometer in a 90% H_2_O/10%D_2_O solvent using excitation sculpting for water suppression. Circular Dichroism (CD): The CD spectra were measured using a Chirascan V100 Circular Dichroism spectrometer (Applied Photophysics Ltd, UK). Isothermal Titration Calorimetry (ITC): All calorimetric data originates from Nano ITC (TA Instruments, USA). Cytometry instrument: The flow cytometry experiments were all conducted in BD FACSVerse (BD, USA). Confocal laser scanning microscope (CLSM): All confocal imaging assays were finished by using the Leica TCS SP8 confocal microscope (Leica, Germany) or Nikon CSU‐W1 SoRa (Nikon, Japan). UV–vis Spectrophotometer: All UV analysis experiments were performed on Agilent Cary 100 (Agilent, USA). In vivo fluorescence imaging: All in vivo fluorescence imaging was performed using PerkinElmer IVIS Spectrum (PerkinElmer, USA).

### Synthesis of Glyconucleic Acid Modules

The glyconucleic acid modules were prepared using a four‐step synthetic route. Four natural glycoligands, namely galactose (Gal), N­acetyl galactosamine (GalNAc), glucose (Glu), and mannose (Man), were chosen as raw materials to form glyconucleic acid modules. In the first step, fully acetylated sugars were modified with a terminal alkyne portion through a classical Fisher glycosylation reaction using BF_3_∙OEt_2_ as the catalyst. Next, the alkynylated glycosides were connected with the pyridine ring of thymine via a Sonogashira cross‐coupling reaction using Pd(PPh_3_)_4_ as the catalyst at room temperature. Finally, the phosphorite monomer was modified to the deoxyribose site 3 of thymine to obtain the glyconucleic acid modules. The successful synthesis of each compound was confirmed through NMR spectra of hydrogen, carbon, phosphorus, and mass spectrometry. The detailed experimental procedures are provided in the supporting information.

### DNA Solid‐Phase Synthesis of GNAAs

Oligonucleotides (1.0 µmol scale) were automatically synthesized on the Expedite Nucleic Acid Synthesis System using β‐cyanoethylphosphoramidite chemistry and benzimidazolium triflate as the activator. The glycosylated‐modified phosphoramidite was dissolved in anhydrous acetonitrile and loaded onto the machine. The coupling efficiency was monitored by measuring the quantity of trityl cations released. The modified phosphoramidite was then introduced to the ODNs on the resin, and the coupling time was extended. After completing the synthetic cycles, the ODNs were deprotected and cleaved from the resin by treating the resin with aqueous ammonia overnight at room temperature. The ODNs were subsequently desalted using a Sephadex G‐25 cartridge column (NAP‐10), and purified by HPLC. The final purification step was performed by reverse‐phase high‐performance liquid chromatography (RP‐HPLC) using a Hypersil BDS C18 column (5 µm, 250 mm × 4.6 mm) with an eluent consisting of 0.1 m ammonium acetate and CH_3_CN.

### Non‐Denatured Polyacrylamide Gel Electrophoresis (Native‐PAGE)

PAGE analysis was performed in a gel containing 10% acrylamide (29:1, acrylamide/bisacrylamide). The running buffer was 1 × TBE buffer, which contained 89 mm Tris, 89 mm boric acid, and 2 mm EDTA. Each sample solution was loaded onto the gel and then run on an FB‐VE10‐1 electrophoresis unit (Fisher Biotech, 300 V, constant voltage). Gel imaging was carried out after electrophoresis using GelRed staining and UV illumination.

### Circular Dichroism Measurements

GNAAs or unmodified aptamers were dissolved in 1× Tris‐EDTA buffer containing Mg^2+^ (5 mm) to obtain a final concentration of 5 µm in a volume of 200 µL at room temperature. Three scans were performed in the range of 320 to 200 nm using a 1 mm cell, with a data pitch of 0.5 nm, a bandwidth of 1 nm, and a scan speed of 50 nm min^−1^.

### Melting Temperature Measurements (Tm)

GNAAs or unmodified aptamers were dissolved in a buffer solution of 10 mm PIPES (1,4‐Piperazinediethanesulfonic acid), 100 mm NaCl, 10 mm MgCl_2_, pH 7.0 with a final concentration of 1 µm. These samples were heated at a rate of 1 °C min^−1^. Their UV signal at 260 nm was recorded using an ultraviolet spectrophotometer. The midpoint at which light absorption increases is estimated to obtain Tm.

### Molecular Docking

To investigate the interaction between GNAAs and Exo1, molecular dynamics simulations were carried out. Two systems were constructed: one is for 5′ glycosylated Sgc8 (GS01) and one is for unmodified Sgc8. The GROMACS 5.0 software was utilized to perform the molecular dynamics simulations, using the SPCE water model. The binding free energy of the Sgc8 and GS01 to Exo1 was studied in MM‐PBSA in GROMACS software. The interaction between Sgc8 and Exo1 protein is shown in Table S2 (Supporting Information), while the interaction between GS01 and Exo1 protein is shown in Table S3 (Supporting Information).

### NMR of Aptamers

Samples at a concentration of 0.6 mm were dissolved in a buffer consisting of 10 mm sodium phosphate (NaPi, pH 7) with 5 mm MgCl_2_, NMR spectra for Sgc8 or GS01 were collected in a 90% H_2_O/10% D_2_O solvent using excitation sculpting for water suppression at 4 °C. The NMR data of the aptamers were collected on a Bruker AVANCE 700 MHz spectrometer and analyzed using Mestrenova 15.

### Isothermal Titration Calorimetry

In the sample cell, 350 µL of Exo1 at a concentration of 10 µm was added. Aptamer (Sgc8 or GS01) at a concentration of 100 µm, was then titrated into the sample cell at a rate of 2 µL per injection over 300 s, resulting in a total of 20 injections. The stirring speed of the sample cell was set to 300 rpm, and the temperature was maintained at 37 °C. The buffer solution consisted of 67 mm Glycine‐KOH, 6.7 mm MgCl_2_, and 1 mm TCEP (Tris(2‐carboxyethyl) phosphine hydrochloride).

### Cell Lines and Cell Culture

HCT116, MCF‐7, and CCRF‐CEM cells were obtained from ATCC. HCT116 cells were grown in McCoy's 5A medium (Gibco, ThermoFisher, Shanghai, China) supplemented with 10% fetal bovine serum (Gibco, ThermoFisher, Shanghai, China). MCF‐7 cells were cultured in Dulbecco's modified Eagle's medium (Gibco, ThermoFisher, Shanghai, China) supplemented with 10% fetal bovine serum. CCRF‐CEM cells were grown in RPMI‐1640 medium (Gibco, ThermoFisher, Shanghai, China) supplemented with 10% fetal bovine serum. Unless otherwise specified, all cell cultures were incubated in a 5% CO_2_ environment at 37 °C.

### Flow Cytometric Analysis of Binding Effect

The binding affinity of GNAAs was assessed by measuring the dissociation constant (Kd) values. Initially, 5×10^6^ cells were seeded in a 100 mm cell culture dish and cultured for 48 h. Then they were treated with 1 mL of 0.2% EDTA. After being washed three times with 1 mL of washing buffer, cells (5 × 10^5^) were incubated with Cy5‐labeled GNAAs with different concentration gradients (0, 0.1, 0.2, 0.5, 1, 2, 5, 10, 20, 50, 100 nm) in 500 µL of binding buffer on ice for 30 min. Finally, the cells were resuspended in 200 µL of washing buffer for flow cytometric analysis to measure the fluorescence intensity on the cell surface. The average fluorescence intensity was obtained using FlowJo. The equilibrium dissociation constant (Kd) value was calculated by fitting the average fluorescence intensity and concentration to the equation of Y = Bmax × X / (Kd + X) using Origin software (OriginLab, USA).

### Washing Buffer

5 mm MgCl_2_ and 4.5 g L^−1^ glucose in Dulbecco's PBS.

### Binding Buffer

5 mm MgCl_2_, 4.5 g L^−1^ glucose, 0.1 mg mL^−1^ yeast tRNA, and 1 mg mL^−1^ BSA in Dulbecco's PBS.

### Flow Cytometric Analysis of Uptake Effect

A total of 2 × 10^5^ cells were seeded in each well of 12‐well plates and cultured at 37 °C in a humid atmosphere with 5% CO_2_ for 24 h. Following this, the cells were treated with Cy5‐labeled GNAAs (250 nm) and incubated for 0, 4, 8, 12, and 24 h. After enzymatic digestion of the cells, they were washed three times with washing buffer. Finally, the cells were resuspended in 200 µL of washing buffer for flow cytometric analysis to measure fluorescence intensity. The average fluorescence intensity was determined using FlowJo.

### Confocal Microscopy Imaging Analysis of Binding Effect

A total of 1 × 10^5^ cells were seeded per confocal dish and cultured at 37 °C in a humid atmosphere with 5% CO_2_ for 24 h. After washing with a cold washing buffer, the cells were treated with GNAAs (500 nm) for 0.5 h at 4 °C. Then, the cells were washed with the washing buffer three times and imaged with a Nikon CSU‐W1 SoRa (Nikon, Japan).

### Confocal Microscopy Imaging Analysis of Uptake Effect

A total of 1 × 10^5^ cells were seeded per confocal dish and cultured at 37 °C in a humid atmosphere with 5% CO_2_ for 24 h. Following washing with cold washing buffer, the cells were treated with Cy5‐labeled GNAAs (250 nm) for varying amounts of time at 37 °C. Subsequently, the cells were washed with washing buffer three times, stained with Cell Mask Green working solutions (1×) for 10 min and Hoechst 33 342 working solutions (1×) for an additional 10 min. The cells were then washed with PBS three times and imaged using a Leica TCS SP8 confocal microscope (Leica, Germany).

### Colocalization of GNAAs with LysoTracker Green or MitoTracker Green Staining

First, HCT 116 cells were seeded on a confocal dish at a density of 1 × 10^5^ cells per well and incubated overnight. Afterward, the cells were treated with GNAAs (500 nm) for 12 h. Following the treatment, the cells were washed three times with PBS and then stained with LysoTracker Green or MitoTracker Green working solutions (100 nm) for 30 min. Additionally, the cells were stained with Hoechst working solutions (1×) for an additional 10 min. Subsequently, the cells were washed three times with PBS and imaged using a confocal microscope (Leica TCS SP8).

### In Vitro Cytotoxicity Assay of GNAAs

The in vitro cytotoxicity was determined using the Cell Counting Kit‐8 (CCK8, Beyotime) assay with 96‐well plates. Cells were seeded into 96‐well plates at a density of 1 × 10^5^ cells per well and incubated overnight. Subsequently, the cells were treated with GNAAs or unmodified aptamers in the medium. After 4 h, the medium was removed and replaced with a fresh medium containing 10% FBS. The cells were then cultured for 72 h, and cell viability was determined using the CCK‐8 method and measured with the Synergy H1 instrument (BioTek, USA).

### In Vivo Biodistribution of GNAAs

A total of 4‐week‐old female nude mice were purchased from the Animal Laboratory Center of Shanghai Institute of Family Planning Science and housed in an SPF laboratory animal room. The animal experiments were conducted in accordance with the Guidelines for the Care and Use of Research Animals established by Renji Hospital, School of Medicine, Shanghai Jiao Tong University. All animal experiments were approved by the Ethics Committee of Renji Hospital, School of Medicine, Shanghai Jiao Tong University. The approval number is RJ2022‐1117.

For in vivo biodistribution analysis, tumor xenografts were established by subcutaneously injecting HCT 116 cells (5×10^6^) into the lateral flanks of nude mice. When the volume of the xenograft tumors reached 5 mm × 5 mm × 5 mm, the nude mice were randomly divided into four groups (*n* = 5), including Sgc8, GS01, GS02, and GS03 groups. Different groups received corresponding Sgc8 or GNAAs treatment (35 µm, 200 µL). After intravenous injection, the mice were anesthetized and visualized using the IVIS in vivo imaging system at different time points (0, 3 min, 0.5, 1, 2, 4, 6, and 24 h). The fluorescence intensity of the tumor region was evaluated in the IVIS software. For the ex vivo biodistribution imaging, the mice were sacrificed after administration at different time points (0.5, 24 h), and major tissues (heart, liver, spleen, lung, kidneys, and tumor) were dissected and rinsed with saline. The fluorescent images of tissue dissections were immediately recorded by the imaging system.

To investigate toxicity, the tumor‐bearing nude mice were divided into four groups, including Sgc8, GS01, GS02, and GS03 groups. At 0.5 and 24 h, major tissues (heart, liver, spleen, lung, kidney, and tumor) were collected to assess toxicity by H&E staining, respectively.

### Statistical Analysis

Quantitative data were presented as mean ± standard deviation (SD). Statistical differences were evaluated using One‐way ANOVA analysis. All tests were analyzed using statistical software (SPSS, version 19.0). A *p*‐value of less than 0.05 was considered statistically significant (^*^ *p* < 0.05, ^**^ *p* < 0.01, ^***^ *p* < 0.001).

## Conflict of Interest

The authors declare no conflict of interest.

## Supporting information



Supporting Information

## Data Availability

The data that support the findings of this study are available from the corresponding author upon reasonable request.
