# Supplementary material for: Molecular Programming Design of Glyconucleic Acid Aptamer with High Stability
Source: Adv Sci (Weinh). 2024 Dec 4;12(4):2408168. doi: 10.1002/advs.202408168 (PMC11775523; doi:10.1002/advs.202408168)
Supplement: Supplementary file 1 — Supporting Information [file ADVS-12-2408168-s001.docx]

Supplementary Information

**Molecular Programming Design of Glyconucleic Acid Aptamer with High Stability**

*Yongqi Han, Rongjun Zhang, Hong-Liang Bao, Mei Yang, Yuan Gao, Xiaobo Gao, Ruowen Wang, Weihong Tan*, Ding-Kun Ji**

Y. Han, R. Zhang, H. -L. Bao, M. Yang, Y. Gao, R. Wang, W. Tan, D.-K. Ji

Institute of Molecular Medicine (IMM), Renji Hospital, State Key Laboratory of Oncogenes and Related Genes, Shanghai Jiao Tong University School of Medicine, Shanghai 200240, China.

Email: Dr. W. Tan, tan@hnu.edu.cn; Dr. D.-K. Ji. dingkunji@sjtu.edu.cn.

Y. Han

College of Chemistry and Materials Science, Shanghai Normal University, Shanghai 200234, China.

W. Tan

Zhejiang Cancer Hospital, Hangzhou Institute of Medicine (HIM), The Chinese Academy of Sciences, Hangzhou, Zhejiang 310022, China.

W. Tan

Molecular Science and Biomedicine Laboratory (MBL), State Key Laboratory of Chemo/Biosensing and Chemometrics, College of Chemistry and Chemical Engineering, College of Biology, Aptamer Engineering Center of Hunan Province, Hunan University Changsha, Hunan, 410082, China.

X. Gao

Department of Anatomy and Physiology, Shanghai Jiao Tong University School of Medicine, Shanghai 200025, China

**Content list：**

**S1. Additional experimental procedures**

**S2.** **Additional Table S1-S4**

**S3. Additional Figure S1-S42**

**S1. Additional Experimental Procedures**

**1. Synthesis of 5-Iodo-5’-dimethoxytrityl-2’-deoxyuridine (UD)**

5-Iodo-2'-deoxyuridine (5.00 g, 2.74 mmol) was dissolved into 45 mL anhydrous pyridine with DMTr-C1 (7.20 g, 2.74 mmol) under Ar gas protection. The reaction was monitored by TLC. After 5-Iodo-2'-deoxyuridine was completely reacted, 3 mL methanol was added to quench the reaction. Then the solvent was removed through rotary evaporator to obtain the crude product. The final product was obtained by column chromatography (CH_2_Cl_2_: EA=10:1-1:1-1:1.5-1:3 and 1% TEA was added). The final compound **UD** (4.83 g, 51%) is a pale-yellow powder.

**2. Synthesis of 1-(5-Hexyn-1-yl)-2,3,4,6-tetra-O-acetyl-β-D-Galactopyranoside (2a)**

To a solution of **1a** (5.00 g, 12.81 mmol) in anhydrous CH_2_Cl_2_, 5-Hexyn-1-ol (2.51 g, 25.62 mmol) and boron trifluoride etherate (3.63 g, 25.62 mmol) was slowly added in an ice bath. The reaction mixture was stirred in ice bath for 30 minutes with an argon atmosphere and then stirred at room temperature overnight. The reaction was quenched by adding saturated NaHCO_3_ solution (10 mL), then extracted with CH_2_Cl_2_ (3 × 10 mL). The combined organic layers were collected and dried with anhydrous Na_2_SO_4_, and then concentrated in vacuo. The residue was purified by flash chromatography to give the compound **2a** (2.20 g, 40 %) as a colorless oily liquid. ^1^H NMR (400 MHz, Chloroform-*d*) δ 5.39 (dd, *J* = 3.5, 1.2 Hz, 1H), 5.21 (dd, *J* = 10.5, 7.9 Hz, 1H), 5.02 (dd, *J* = 10.5, 3.4 Hz, 1H), 4.46 (d, *J* = 8.0 Hz, 1H), 4.23 – 4.06 (m, 3H), 3.98 – 3.86 (m, 2H), 3.54 – 3.49 (m, 1H), 2.23 – 2.19 (m, 2H), 2.15 (s, 3H), 2.07 (s, 2H), 2.05 (s, 3H), 1.99 (s, 3H), 1.95 (t, *J* = 2.7 Hz, 1H), 1.74 – 1.68 (m, 2H), 1.65 – 1.53 (m, 2H). ^13^C NMR (101 MHz, Chloroform-*d*) δ 170.42, 170.37, 170.30, 170.21, 170.14, 169.89, 169.42, 168.93, 101.32, 89.71, 84.08, 70.94, 70.60, 69.47, 68.88, 68.75, 68.60, 67.41, 67.35, 67.06, 66.43, 61.28, 61.24, 28.38, 24.80, 20.89, 20.76, 20.69, 20.66, 20.64, 20.61, 20.55, 18.03.

**3. Synthesis of 5-[1-(2,3,4,6-tetra-O-acetyl-β-D-Galactopyranosyl)-5-hexyn-6-yl]-2’-deoxy-5’-O-(4,4’-dimethoxytrityl)uridine (3a)**

To a solution of **UD** (2.56 g, 3.89 mmol), Pd (PPh_3_)_4_ (0.90 g, 0.78 mmol), and CuI (0.30 g, 1.56 mmol) in DMF (20 mL), TEA (0.54 mL, 3.89 mmol.) was added. The reaction mixture was stirred at room temperature for 10 minutes. A solution of compound **2a** (2.00 g, 4.67 mmol) in DMF (5 mL) was added dropwise to the reaction mixture over 1 hour. The reaction mixture was stirred at room temperature overnight. After concentration in vacuo, the obtained crude was dissolved in ethyl acetate (200 mL) and washed with brine (3 x 50 mL). The organic layer was dried with anhydrous Na_2_SO_4_ and concentrated in vacuo. Flash column chromatography eluting with a gradient of CH_2_Cl_2_ : EA (10:1) followed by CH_2_Cl_2_:EA (3:1) provided compound **3a** (1.60 g, 43 %) as a pale-yellow powder.^1^H NMR (400 MHz, DMSO-*d*_6_) δ 11.62 (s, 1H), 7.88 (s, 1H), 7.45 – 7.38 (m, 2H), 7.34 – 7.28 (m, 6H), 7.26 – 7.21 (m, 1H), 6.91 – 6.87 (m, 4H), 6.13 (t, *J* = 6.7 Hz, 1H), 5.26 (dd, *J* = 3.5, 1.2 Hz, 1H), 5.16 (dd, *J* = 10.4, 3.6 Hz, 1H), 4.93 (dd, *J* = 10.5, 8.0 Hz, 1H), 4.65 (d, *J* = 8.0 Hz, 1H), 4.30 – 4.27 (m, 1H), 4.23 – 4.15 (m, 1H), 4.08 – 4.00 (m, 3H), 3.96 – 3.87 (m, 2H), 3.74 (s, 6H), 3.67 – 3.62 (m, 1H), 3.23 – 3.19 (m, 1H), 3.15 – 3.12 (m, 1H), 2.30 – 2.22 (m, 1H), 2.21 – 2.15 (m, 2H), 2.11 (s, 3H), 1.99 (s, 6H), 1.92 (s, 3H), 1.50 – 1.38 (m, 2H), 1.32 – 1.23 (m, 2H), 1.18 (t, *J* = 7.1 Hz, 1H). ^13^C NMR (101 MHz, DMSO-*d6*) δ 170.44, 170.36, 170.01, 169.60, 162.20, 158.55, 149.84, 145.20, 142.46, 136.03, 135.86, 130.16, 130.12, 128.36, 128.07, 127.14, 113.68, 100.38, 99.77, 93.56, 86.32, 85.23, 72.88, 70.89, 70.69, 70.25, 69.14, 68.91, 67.81, 64.07, 61.77, 60.23, 55.49, 28.58, 24.82, 21.23, 20.95, 20.91, 20.85, 20.82, 18.81, 14.56.

**4. Synthesis of 5-(1-(2,3,4,6-tetra-O-acetyl-β-D-Galactopyranosyl)-5-hexyn-6-yl)-2’-deoxy-5’-O-(4,4’-dimethoxytrityl)uridine-3’-O-(2-cyanoethyl-N,N’-diisopropyl)-phosphoramidite (4a)**

To a solution of compound **3a** (100 mg, 0.10 mmol) and 2-cyanoethyl-N,N-diisopropylchlorophosphamide (110 mg, 0.42 mmol) , DIEA (0.15 mL, 0.84 mmol.) in anhydrous CH_2_Cl_2_ added in an ice bath under an argon atmosphere. The reaction mixture was stirred at room temperature for 2 hours. Then the reaction mixture was quenched by saturated sodium bicarbonate solution (1 mL) and then extracted with CH_2_Cl_2_ (3 × 10 mL). The combined organic layers were collected and dried with anhydrous Na_2_SO_4_, and then concentrated in vacuo. The obtained crude product was purified by silica column chromatography eluted with (CH_2_Cl_2_:EA,100:1 to 20:1 to 10:1) to give the compound **4a** (75.8 mg, 63 %) as a yellow powder.^1^H NMR (400 MHz, DMSO-*d*_6_) δ 11.63 (s, 1H), 7.92 (s, 1H), 7.82 (d, *J* = 9.2 Hz, 1H), 7.45 – 7.37 (m, 2H), 7.33 – 7.27 (m, *J* = 8.5, 5.8 Hz, 5H), 7.26 – 7.20 (m, 1H), 6.88 (dd, *J* = 8.6, 4.4 Hz, 4H), 6.12 (q, *J* = 6.9 Hz, 1H), 5.22 – 5.20 (m, 1H), 4.97 (dd, *J* = 11.3, 3.4 Hz, 1H), 4.52 – 4.46 (m, 2H), 4.11 – 3.98 (m, 5H), 3.93 – 3.80 (m, 2H), 3.74 (s, 6H), 3.65 – 3.56 (m, 3H), 3.55 – 3.46 (m, 1H), 3.27 – 3.24 (m, 1H), 2.76 (t, *J* = 5.9 Hz, 1H), 2.62 (dt, *J* = 18.8, 5.9 Hz, 2H), 2.47 –2.38 (m, 1H), 2.34 – 2.25 (m, 1H), 2.33 – 2.29 (m, 2H), 2.10 (s, 3H), 1.99 (d, *J* = 2.7 Hz, 4H), 1.90 (s, 3H), 1.77 (s, 3H), 1.49 – 1.40 (m, 2H), 1.34 – 1.26 (m, 2H), 1.21 – 1.16 (m, 2H), 1.14 – 1.08 (m, 8H), 0.98 (d, *J* = 6.8 Hz, 2H). ^13^C NMR (101 MHz, DMSO-*d6*) δ 170.36, 170.00, 169.60, 162.18, 158.59, 149.80, 145.09, 142.78, 135.89, 135.72, 130.14, 130.09, 128.34, 128.04, 127.16, 120.19, 119.40, 113.65, 100.38, 99.87, 86.36, 85.33, 72.90, 70.68, 70.25, 69.13, 68.91, 67.81, 61.76, 59.32, 58.93, 58.75, 57.00, 55.48, 52.46, 46.15, 43.09, 42.97, 28.57, 24.80, 24.73, 24.67, 24.59, 21.45, 20.94, 20.91, 20.84, 20.81, 20.31, 20.24, 18.80, 17.82, 11.70. ^31^P NMR (162 MHz, DMSO-*d6*) δ 147.50, 147.11.

**5. Synthesis of 1-(5-Hexyn-1-yl)-2,3,4,6-tetra-O-acetyl-α-D-Mannopyranoside (2b)**

Compound **2b** is synthesized according to the preparation process of compound 2a. From **1b** (3.00 g, 7.69 mmol) and 5-Hexyn-1-ol (1.50 g, 15.37 mmol) and boron trifluoride etherate (2.18 g, 15.37 mmol), column chromatography (CH_2_Cl_2_: EA, 100:1 to 10:1, v/v) afforded compound **2b** (2.00 g, 61 %) as a colorless oily liquid. ^1^H NMR (400 MHz, DMSO-*d*_6_) δ 5.13 – 5.07 (m, 3H), 4.87 (d, *J* = 1.5 Hz, 1H), 4.15 (dd, *J* = 12.2, 5.4 Hz, 1H), 4.06 (t, *J* = 2.9 Hz, 1H), 3.94 – 3.90 (m, 1H), 3.67 – 3.62 (m, 1H), 3.51 – 3.45 (m, 1H), 2.78 (t, *J* = 2.7 Hz, 1H), 2.23 – 2.18 (m, 2H), 2.11 (s, 3H), 2.03 (s, 6H), 1.94 (s, 3H), 1.70 – 1.62 (m, 2H), 1.55 – 1.47 (m, 2H). ^13^C NMR (101 MHz, DMSO-*d*_6_) δ 170.82, 170.52, 170.15, 170.12, 170.00, 105.14, 97.01, 84.74, 71.87, 71.74, 71.61, 69.21, 69.16, 68.35, 67.37, 65.85, 62.49, 60.57, 60.23, 31.98, 30.83, 29.48, 28.34, 25.17, 25.11, 21.23, 21.08, 20.97, 20.91, 20.88, 18.00, 17.87, 14.54, 14.43. TOF-MS (ESI): m/z 451.1574 [M + Na]^+^

**6. Synthesis of 5-[1-(2,3,4,6-tetra-O-acetyl-α-D-Mannopyranoyl)-5-hexyn-6-yl]-2’-deoxy-5’-O-(4,4’-dimethoxytrityl)uridine (3b)**

Compound **3b** is synthesized according to the preparation process of compound **3a**. From **2b** (2.00 g, 4.67 mmol), Pd (PPh_3_)_4_ (0.90 g, 0.78 mmol), CuI (0.30 g, 1.56 mmol), TEA (0.54 mL, 3.89 mmol) and UD (2.55 g, 3.89 mmol) in DMF (60 mL), column chromatography (CH_2_Cl_2_/MeOH, 100/1 to 80:1, v/v) afforded 3b (2.83 g, 76 %) as a pale-yellow powder. ^1^H NMR (400 MHz, DMSO-*d*_6_) δ 11.63 (s, 1H), 7.90 (s, 1H), 7.42 (d, *J* = 7.4 Hz, 2H), 7.34 – 7.29 (m, 6H), 7.23 (t, *J* = 7.2 Hz, 1H), 6.89 (d, *J* = 8.6 Hz, 4H), 6.13 (t, *J* = 6.7 Hz, 1H), 5.77 (s, 1H), 5.35 (d, *J* = 4.4 Hz, 1H), 5.15 – 5.05 (m, 3H), 4.85 (d, *J* = 1.4 Hz, 1H), 4.32 – 4.29 (m, 1H), 4.14 (dd, *J* = 12.3, 5.2 Hz, 1H), 4.03 (dd, *J* = 12.2, 2.5 Hz, 1H), 3.96 – 3.86 (m, 2H), 3.74 (s, 6H), 3.58 – 3.51 (m, 1H), 3.42 – 3.36 (m, 1H), 3.22 (dd, *J* = 10.6, 5.0 Hz, 1H), 3.13 – 3.10 (m, 1H), 2.29 – 2.22 (m, 1H), 2.18 (t, *J* = 7.1 Hz, 2H), 2.11 (s, 3H), 2.01 (d, *J* = 3.4 Hz, 6H), 1.94 (s, 3H), 1.57 – 1.50 (m, 2H), 1.40 – 1.28 (m, 2H). ^13^C NMR (101 MHz, DMSO-*d6*) δ 170.52, 170.14, 170.10, 169.99, 162.23, 158.53, 149.84, 145.20, 142.53, 136.02, 135.85, 130.13, 128.36, 128.07, 127.13, 113.67, 99.75, 97.02, 93.53, 86.32, 85.23, 70.90, 69.20, 69.17, 68.31, 67.36, 65.83, 64.07, 62.45, 55.48, 55.40, 28.48, 25.10, 21.09, 20.96, 20.91, 20.89, 18.94. TOF-MS (ESI): m/z 957.3642 [M + H]^+^, m/z 979.3464 [M + Na]^+^

**7. Synthesis of 5-(1-(2,3,4,6-tetra-O-acetyl-α-D-Mannopyranoyl)-5-hexyn-6-yl)-2’-deoxy-5’-O-(4,4’-dimethoxytrityl)uridine-3’-O-(2-cyanoethyl-N,N’-diisopropyl)-phosphoramidite (4b)**

Compound **4b** is synthesized according to the preparation process of compound **4a**. From **3b** (1.00 g, 1.00 mmol) DIEA (1.46 mL, 8.40 mmol) and 2-cyanoethyl-N, N-diisopropylchlorophosphamide (1.10 g, 4.20 mmol), column chromatography (CH_2_Cl_2_: EA, 100:1 to 20:1 to 10:1) afforded 4b (1.01 g, 83 %) as a yellow powder. ^1^H NMR (400 MHz, DMSO-*d*_6_) δ 11.65 (s, 1H), 7.93 (s, 1H), 7.45 – 7.38 (m, 2H), 7.35 – 7.26 (m, 6H), 7.25 – 7.20 (m, 1H), 6.91 – 6.84 (m, 4H), 6.13 (t, *J* = 6.7 Hz, 1H), 5.11 – 5.09 (m, 3H), 4.85 (d, *J* = 1.5 Hz, 1H), 4.53 – 4.47 (m, 1H), 4.14 (dd, *J* = 12.2, 5.2 Hz, 1H), 4.07 – 4.02 (m, 2H), 4.01 – 3.97 (m, 1H), 3.92 – 3.88 (m, 1H), 3.73 (s, 6H), 3.59 – 3.45 (m, 3H), 3.42 – 3.36 (m, 1H), 3.28 – 3.24 (m, 1H), 3.19 – 3.15 (m, 1H), 2.76 (t, *J* = 5.9 Hz, 2H), 2.48 – 2.31 (m, 2H), 2.21 (t, *J* = 7.0 Hz, 2H), 2.11 (s, 3H), 2.01 (d, *J* = 2.4 Hz, 4 H), 1.99 (d, *J* = 1.8 Hz, 2H), 1.94 (s, 3H), 1.58 – 1.50 (m, 2H), 1.39 – 1.29 (m, 2H), 1.18 (t, *J* = 7.1 Hz, 2H), 1.11 (d, *J* = 6.7 Hz, 5H), 0.98 (d, *J* = 6.7 Hz, 5H), 0.88 (t, *J* = 7.4 Hz, 1H). ^13^C NMR (101 MHz, DMSO-*d*_6_) δ 170.52, 170.14, 170.09, 169.98, 162.19, 158.58, 149.81, 145.09, 142.86, 135.89, 135.71, 130.14, 130.09, 128.34, 128.04, 127.15, 119.42, 113.64, 99.85, 97.02, 93.60, 86.36, 85.34, 84.92, 73.40, 73.22, 72.96, 69.21, 69.17, 68.31, 67.36, 65.84, 63.96, 63.71, 62.46, 60.23, 58.93, 58.75, 55.47, 43.06, 42.94, 30.63, 28.46, 25.12, 24.80, 24.73, 24.67, 24.59, 21.23, 21.19, 21.08, 20.95, 20.90, 20.88, 20.31, 20.24, 19.08, 18.94, 14.55, 14.03. ^31^P NMR (162 MHz, DMSO-*d*_6_) δ 147.43, 147.05. TOF-MS (ESI): m/z 1179.4547 [M + H]^+^.

**8. Synthesis of 1-(5-Hexyn-1-yl)-2,3,4,6-tetra-O-acetyl-β-D-Glucopyranoside (2c)**

Compound **2c** is synthesized according to the preparation process of compound **2a**. From **1c** (5.00 g, 12.81 mmol) and 5-Hexyn-1-ol (2.51 g, 25.61 mmol), column chromatography (PE/EA, 10/1) afforded **2c** (2.34 g, 43 %) as a colorless oily liquid. ^1^H NMR (400 MHz, Chloroform-*d*) δ 5.21 (t, *J* = 9.5 Hz, 1H), 5.09 (t, *J* = 9.6 Hz, 1H), 4.99 (dd, *J* = 9.6, 8.0 Hz, 1H), 4.50 (d, *J* = 8.0 Hz, 1H), 4.27 (dd, *J* = 12.3, 4.7 Hz, 1H), 4.16 – 4.12 (m, 1H), 3.94 – 3.89 (m, 1H), 3.72 – 3.67 (m, 1H), 3.54 – 3.48 (m, 1H), 2.23 – 2.18 (m, 2H), 2.09 (s, 3H), 2.06 (s, 3H), 2.03 (s, 3H), 2.01 (s, 3H), 1.95 (t, *J* = 2.6 Hz, 1H), 1.74 – 1.66 (m, 2H), 1.63 – 1.54 (m, *J* = 8.7, 6.4 Hz, 2H). ^13^C NMR (101 MHz, Chloroform-*d*) δ 170.73, 170.35, 169.43, 169.34, 100.78, 99.24, 84.06, 73.76, 72.80, 71.73, 71.25, 70.33, 69.65, 69.44, 69.09, 68.59, 68.39, 65.37, 61.93, 60.41, 28.32, 24.76, 20.91, 20.77, 20.68, 20.65, 20.62, 17.99, 15.04, 14.20. TOF-MS (ESI): m/z 451.1583 [M + Na]^+^.

**9. Synthesis of 5-[1-(2,3,4,6-tetra-O-acetyl-β-D-Glucopyranoyl)-5-hexyn-6-yl]-2’-deoxy-5’-O-(4,4’-dimethoxytrityl) uridine (3c)**

Compound **3c** is synthesized according tothe preparation process of compound **3b**. From **2c** (2.60 g, 6.10 mmol), Pd (PPh_3_)_4_ (1.17 g, 1.01 mmol), CuI (0.39 g, 2.02 mmol), TEA (0.70 mL, 5.06 mmol) and **UD** (3.32 g, 5.06 mmol) in DMF (60 mL), column chromatography (CH_2_Cl_2_/MeOH, 100/1 to 80:1, v/v) afforded **3c** (3.10 g, 64 %) as a pale-yellow powder. ^1^H NMR (400 MHz, DMSO-*d*_6_) δ 11.62 (s, 1H), 7.88 (s, 1H), 7.41 (d, *J* = 7.4 Hz, 2H), 7.33 – 7.28 (m, 6H), 7.23 (t, *J* = 7.1 Hz, 1H), 6.91 – 6.87 (m, 4H), 6.12 (t, *J* = 6.7 Hz, 1H), 5.36 – 5.31 (m, 1H), 5.29 – 5.24 (m, *J* = 9.6, 4.9 Hz, 1H), 4.89 (t, *J* = 9.6 Hz, 1H), 4.77 – 4.72 (m, 2H), 4.28 (s, 1H), 4.17 (dd, *J* = 12.1, 4.8 Hz, 1H), 4.00 – 3.96 (m, 1H), 3.93 – 3.90 (m, 1H), 3.74 (s, 6H), 3.67 – 3.62 (m, 1H), 3.39 – 3.36 (m, 1H), 3.23 – 3.19 (m, 1H), 3.12 (dd, *J* = 10.6, 2.8 Hz, 1H), 2.29 – 2.18 (m, 2H), 2.13 (t, *J* = 7.1 Hz, 2H), 2.00 (s, 3H), 1.98 (s, 3H), 1.97 (s, 3H), 1.94 (s, 3H), 1.48 – 1.41 (m, 2H), 1.32 – 1.22 (m, 3H).  ^13^C NMR (101 MHz, DMSO-*d*_6_) δ 170.52, 170.03, 169.76, 169.49, 162.20, 158.54, 149.83, 145.21, 142.47, 136.02, 135.85, 134.10, 133.96, 130.50, 130.16, 130.12, 129.18, 129.10, 128.36, 128.06, 127.13, 113.68, 99.86, 93.55, 86.32, 85.24, 72.87, 72.49, 71.39, 70.97, 70.89, 68.97, 68.69, 64.07, 62.24, 55.49, 55.39, 28.56, 24.82, 20.95, 20.86, 20.79, 20.76, 18.80. TOF-MS (ESI): m/z 957.3643 [M + H]^+^, 979.3488 [M + Na]^+^.

**10. Synthesis of 5-(1-(2,3,4,6-tetra-O-acetyl-β-D-Glucopyranoyl)-5-hexyn-6-yl)-2’-deoxy-5’-O-(4,4’-dimethoxytrityl)uridine-3’-O-(2-cyanoethyl-N,N’-diisopropyl)-phosphoramidite (4c)**

Compound **4c** is synthesized according to the preparation process of compound **4a**. From **3c** (1.00 g, 1.00 mmol), DIEA (1.46 mL, 8.40 mmol) and 2-cyanoethyl-N, N-diisopropylchlorophosphamide (1.10 g, 4.20 mmol), column chromatography (CH_2_Cl_2_: EA, 100:1 to 20:1, v/v) afforded compound **4c** (0.81 g, 67 %) as a pale-yellow powder. ^1^H NMR (400 MHz, DMSO-*d*_6_) δ 11.62 (s, 1H), 7.91 (s, 1H), 7.43-7.39 (m, 2H), 7.30 – 7.26 (m, 6H), 7.24 – 7.20 (m, 1H), 6.87 (dd, *J* = 8.8, 4.3 Hz, 4H), 6.11 (q, *J* = 7.0 Hz, 1H), 5.31 – 5.21 (m, 2H), 4.88 (t, *J* = 9.6 Hz, 1H), 4.75 – 4.73 (m, 2H), 4.53 – 4.47 (m, 1H), 4.17 (dd, *J* = 12.2, 4.9 Hz, 1H), 4.09 – 4.05 (m, 3H), 3.99 – 3.95 (m, 2H), 3.73 (s, 5H), 3.65 – 3.55 (m, 4H), 3.54 – 3.46 (m, 2H), 3.27 – 3.22 (m, 2H), 3.19 – 3.16 (m, 1H), 2.76 (t, *J* = 5.8 Hz, 1H), 2.64 (t, *J* = 5.9 Hz, 1H), 2.59 (t, *J* = 6.0 Hz, 1H), 2.46 – 2.36 (m, 1H), 2.35 – 2.27 (m, 1H), 2.18 – 2.10 (m, 2H), 2.00 (s, 3H), 1.98 (s, 3H), 1.97 (s, 3H), 1.93 (s, 3H), 1.47 – 1.39 (m, 2H), 1.35 – 1.26 (m, 2H), 1.13 – 1.06 (m, 9H), 0.97 (d, *J* = 6.5 Hz, 2H). ^13^C NMR (101 MHz, DMSO-*d*_6_) δ 169.98, 158.57, 130.13, 128.35, 128.04, 113.65, 97.02, 69.20, 68.31, 67.37, 65.85, 62.46, 60.24, 55.50, 53.97, 52.49, 46.76, 46.14, 28.46, 25.11, 21.10, 20.97, 20.92, 20.90, 19.28, 18.93, 14.56, 12.99, 9.13, 7.72. TOF-MS (ESI): m/z 1179.4565 [M + Na]^+^.

**11. Synthesis of 1-(5-Hexyn-1-yl)-2-Acetamido**-**3,4,6-tri-O-acetyl-β-D-Galactopyranoside (2d)**

Compound **2d** is synthesized according to the preparation process of compound **2a**. From **1d** (5.00 g, 12.81 mmol) and 5-Hexyn-1-ol (2.51 g, 25.62 mmol) and boron trifluoride etherate (3.63 g, 25.62 mmol), column chromatography (CH_2_Cl_2_: EA, 100:1 to 10:1, v/v) afforded compound 2d (4.48 g, 82 %) as a colorless solid. ^1^H NMR (400 MHz, Chloroform-*d*) δ 5.44 (d, *J* = 8.7 Hz, 1H), 5.38 (d, *J* = 3.3 Hz, 1H), 5.32 (dd, *J* = 11.2, 3.3 Hz, 1H), 4.73 (d, *J* = 8.4 Hz, 1H), 4.22 – 4.12 (m, 2H), 4.02 – 3.91 (m, 3H), 3.57 – 3.52 (m, 1H), 2.26 – 2.20 (m, 2H), 2.17 (s, 3H), 2.07 (s, 3H), 2.03 (s, 3H), 1.99 (s, 3H), 1.77 – 1.69 (m, 2H), 1.66 – 1.58 (m, 2H), 1.34 – 1.27 (m, 1H). ^13^C NMR (101 MHz, Chloroform-*d*) δ 170.50, 170.46, 170.30, 100.93, 84.27, 70.65, 69.87, 69.23, 68.57, 66.81, 61.47, 51.83, 28.37, 24.89, 23.49, 20.73, 20.71, 18.08. TOF-MS (ESI): m/z 428.1919 [M + H]^+^ m/z 450.1736 [M + Na]^+^.

**12. Synthesis of 5-[1-(2-Acetamido**-**3,4,6-tri-O-acetyl-β-D-Galactopyranosyl)-5-hexyn-6-yl]-2’-deoxy-5’-O-(4,4’-dimethoxytrityl) uridine (3d)**

Compound **3d** is synthesized according to the preparation process of compound **3a**. From **2d** (4.48 g, 10.48 mmol), Pd (PPh_3_)_4_ (2.02 g, 1.75 mmol), CuI (0.67 g, 3.49 mmol), TEA (1.67 mL, 8.73 mmol) and UD (5.73 g, 8.73 mmol) in DMF (100 mL), column chromatography (CH_2_Cl_2_/MeOH, 100/1 to 80:1, v/v) afforded 3b (2.83 g, 76 %) as a pale-yellow powder. ^1^H NMR (400 MHz, DMSO-*d*_6_) δ 11.63 (s, 1H), 7.88 (s, 1H), 7.43 – 7.40 (m, 2H), 7.34 – 7.27 (m, 6H), 7.26 – 7.20 (m, 2H), 6.90 – 6.87 (m, 4H), 6.12 (t, *J* = 6.7 Hz, 1H), 5.34 (d, *J* = 4.4 Hz, 1H), 5.30 – 5.23 (m, 1H), 4.89 (t, *J* = 9.7 Hz, 1H), 4.77 – 4.72 (m, 2H), 4.32 – 4.25 (m, 1H), 4.22 – 4.12 (m, 1H), 3.99 – 3.96 (m, 1H), 3.93 – 3.90 (m, 1H), 3.74 (s, 6H), 3.67 – 3.62 (m, 1H), 3.39 – 3.35 (m, 2H), 3.23 – 3.19 (m, 1H), 3.14 – 3.10 (m, 1H), 2.33 – 2.21 (m, 2H), 2.13 (t, *J* = 7.1 Hz, 2H), 2.00 (s, 3H), 1.98 (s, 3H), 1.97 (s, 3H), 1.94 (s, 3H), 1.46 (m, 2H), 1.28 (m, 2H). ^13^C NMR (101 MHz, DMSO-*d*_6_) δ 162.24, 158.53, 149.84, 145.21, 142.45, 136.07, 135.79, 130.18, 130.11, 128.37, 128.05, 127.12, 113.69, 113.66, 99.83, 93.88, 86.32, 85.23, 72.69, 70.92, 64.11, 60.58, 55.48, 55.40, 32.16, 25.13, 19.08. TOF-MS (ESI): m/z 978.3638 [M + Na]^+^.

**13. Synthesis of 5-(1-(2-Acetamido**-**3,4,6-tri-O-acetyl-β-D-Galactopyranosyl)-5-hexyn-6-yl)-2’-deoxy-5’-O-(4,4’-dimethoxytrityl)uridine-3’-O-(2-cyanoethyl-N,N’-diisopropyl)-phosphoramidite (4d)**

Compound **4d** is synthesized according to the preparation process of compound **4a**. From **3d** (1.00 g, 1.00 mmol), DIEA (1.46 mL, 8.40 mmol) and 2-cyanoethyl-N, N-diisopropylchlorophosphamide (1.10 g, 4.20 mmol), column chromatography (CH_2_Cl_2_: EA, 100:1 to 20:1 to 10:1) afforded 4d (0.50 g, 41 %) as a yellow powder. ^1^H NMR (400 MHz, DMSO-*d*_6_) δ 11.63 (s, 1H), 7.92 (s, 1H), 7.82 (d, *J* = 9.2 Hz, 1H), 7.44 – 7.39 (m, 2H), 7.34 – 7.27 (m, 6H), 7.26 – 7.20 (m, 1H), 6.90 – 6.86 (m, 4H), 6.12 (q, *J* = 7.0 Hz, 1H), 5.25 – 5.17 (m, 2H), 4.97 (dd, *J* = 11.2, 3.4 Hz, 1H), 4.55 – 4.46 (m, 2H), 4.08 (q, *J* = 3.8 Hz, 1H), 3.91 – 3.84 (m, 1H), 3.74 (s, 6H), 3.65 – 3.57 (m, 4H), 3.56 – 3.46 (m, 2H), 3.29 – 3.20 (m, 2H), 3.11 – 3.00 (m, 2H), 2.76 (t, *J* = 5.9 Hz, 1H), 2.65 (t, *J* = 5.9 Hz, 1H), 2.60 (t, *J* = 6.0 Hz, 1H), 2.47 – 2.40 (m, 1H), 2.33 – 2.28 (m, 1H), 2.19 – 2.12 (m, 2H), 2.10 (s, 3H), 1.99 (s, 3H), 1.90 (s, 3H), 1.77 (s, 3H), 1.47 – 1.40 (m, 2H), 1.33 – 1.26 (m, 2H), 1.14 – 1.08 (m, 9H), 1.00 – 0.97 (m, 2H). ^13^C NMR (101 MHz, DMSO-*d*_6_) δ 170.48, 170.39, 170.12, 169.73, 162.20, 158.58, 149.80, 145.10, 142.62, 135.89, 135.78, 130.15, 128.34, 128.08, 127.18, 120.19, 119.24, 113.65, 101.41, 99.86, 93.71, 86.42, 85.33, 72.84, 70.92, 70.28, 68.67, 67.17, 61.93, 58.91, 58.72, 57.00, 55.50, 49.78, 46.18, 43.10, 42.98, 28.57, 24.84, 24.77, 24.74, 24.67, 24.59, 23.24, 21.46, 20.98, 20.93, 20.25, 20.19, 18.81. ^31^P NMR (162 MHz, DMSO-*d6*) δ 147.48, 147.09. TOF-MS (ESI): m/z 1178.4683 [M + Na]^+^.

S**2. Additional Table S1-S4**

**Table S1. Oligonucleotide sequences used in the experiments.**

| Oligonucleotides | Sequences ( 5’-3’ ) |
| --- | --- |
| Sgc8 | Cy5-ATCTAACTGCTGCGCCGCCGGGAAAATACTGTACGGTTAGA |
| AS01 | **Y**ATCTAACTGCXGCGCCGCCGGGAAAATACTGTACGGTTAGAT-Cy5 |
| AS02 | Cy5-TATCTAACTGC**Y**GCGCCGCCGGGAAAATACTGTACGGTTAGAT |
| AS03 | Cy5-TATCTAACTGCTGCGCCGCCGGGAAAATACTGTACGGTTAGA**Y** |
| MS01 | **M**ATCTAACTGCXGCGCCGCCGGGAAAATACTGTACGGTTAGAT-Cy5 |
| MS02 | Cy5-TATCTAACTGC**M**GCGCCGCCGGGAAAATACTGTACGGTTAGAT |
| MS03 | Cy5-TATCTAACTGCTGCGCCGCCGGGAAAATACTGTACGGTTAGA**M** |
| DS01 | **D**ATCTAACTGCXGCGCCGCCGGGAAAATACTGTACGGTTAGAT-Cy5 |
| DS02 | Cy5-TATCTAACTGC**D**GCGCCGCCGGGAAAATACTGTACGGTTAGAT |
| DS03 | Cy5-TATCTAACTGCTGCGCCGCCGGGAAAATACTGTACGGTTAGA**D** |
| GS01 | **X**ATCTAACTGCXGCGCCGCCGGGAAAATACTGTACGGTTAGAT-Cy5 |
| GS02 | Cy5-TATCTAACTGC**X**GCGCCGCCGGGAAAATACTGTACGGTTAGAT |
| GS03 | Cy5-TATCTAACTGCTGCGCCGCCGGGAAAATACTGTACGGTTAGA**X** |
| AS1411 | Cy5-GGTGGTGGTGGTTGTGGTGGTGGTGG |
| GA01 | **X**GGTGGTGGTGGTTGTGGTGGTGGTGGT-Cy5 |
| GA02 | Cy5-TGGTGGTGGTGG**X**TGTGGTGGTGGTGGT |
| GA03 | Cy5-TGGTGGTGGTGGTTGTGGTGGTGGTGG**X** |
| MUC1 | Cy5-GCAGTTGATCCTTTGGATACCCTGG |
| GM01 | **X**GCAGTTGATCCTTTGGATACCCTGGT-Cy5 |
| GM02 | Cy5-TGCAGTTGATCC**X**TTGGATACCCTGGT |
| GM03 | Cy5-TGCAGTTGATCCTTTGGATACCCTGG**X** |
| XQ-2d | Cy5-ACTCATAGGGTTAGGGGCTGCTGGCCAGATACTCAGATGGTAGGGTTACTATGAGC |
| GX01 | **X**ACTCATAGGGTTAGGGGCTGCTGGCCAGATACTCAGATGGTAGGGTTACTATGAGCT-Cy5 |
| GX02 | Cy5-TACTCATAGGGTTAGGGGCTGC**X**GGCCAGATACTCAGATGGTAGGGTTACTATGAGCT |
| GX03 | Cy5-TACTCATAGGGTTAGGGGCTGCTGGCCAGATACTCAGATGGTAGGGTTACTATGAGC**X** |
| LSgc8 | Cy5-AACCGATAAAATAAGAGCCGAAGCGTAGATTACCAGTGTAT |
| LA01 | Cy5-AGGTGGTAGTTTCGGGGCCCTCAGTG |
| LM01 | Cy5-CTTGACTGAATCTGTATAAGGAGGC |
| LX01 | Cy5-GAGGCCGTTTGTAGGAAGATTTGGAAAAGGAAATGTAGGAGGTTCTCTTTTCGACG |

**Note:** Y refers to Gal-modified bases, M refers to Man-modified bases, D refers to Glu-modified bases, and X refers to GalN-modified bases in the below sequences.

**Table S2 Interactions between Sgc8 and Exo1**

| **Sgc8** | **Exo1** |
| --- | --- |
| C3 | LYS61 |
| C3 | GLN188 |
| T4 | GLN188 |
| A5 | LYS37 |
| C19 | ARG95 |
| G20 | SER228 |

**Table S3 Interactions between GS01 and Exo1**

| **GS01** | **Exo1** |
| --- | --- |
| T38 | ARG121 |
| C11 | ARG95 |
| T12 | ARG95 |

**Table S4 The thermodynamic data of isothermal titration calorimetry of Sgc8 and GS01 with Exo1**

|  | **Sgc8** | **GS01** |
| --- | --- | --- |
| Kd(M) | 1.000×10^-9^ | 6.620×10^-4^ |
| ΔH(kJ/mol) | 24.24 | -48.08 |
| ΔG(kJ/mol) | -53.44 | -18.88 |
| ΔS(J/mol*K) | 250.5 | -94.16 |

**Note:** Kd: Dissociation constant; ΔH: Enthalpy change; ΔG: Gibbs free energy change; ΔS: Entropy change.

**S3. Additional Figure S1-S42**

NMR and Mass of 1-(5-Hexyn-1-yl)-2,3,4,6-tetra-O-acetyl-α-D-Mannopyranoside (**2b**).


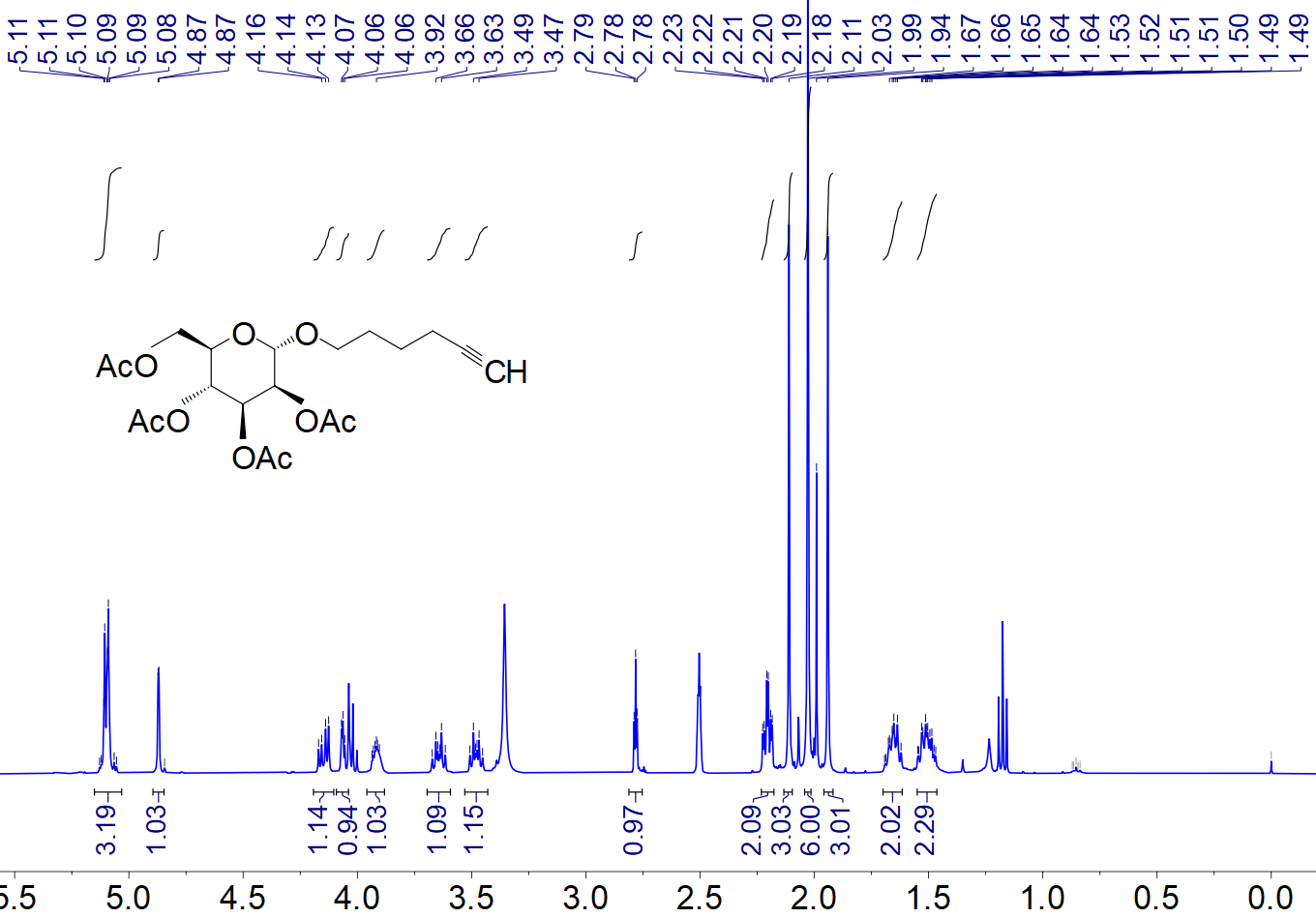


**Figure S1**. ^1^H NMR spectrum of **2b** in DMSO-*d*_6_*.*


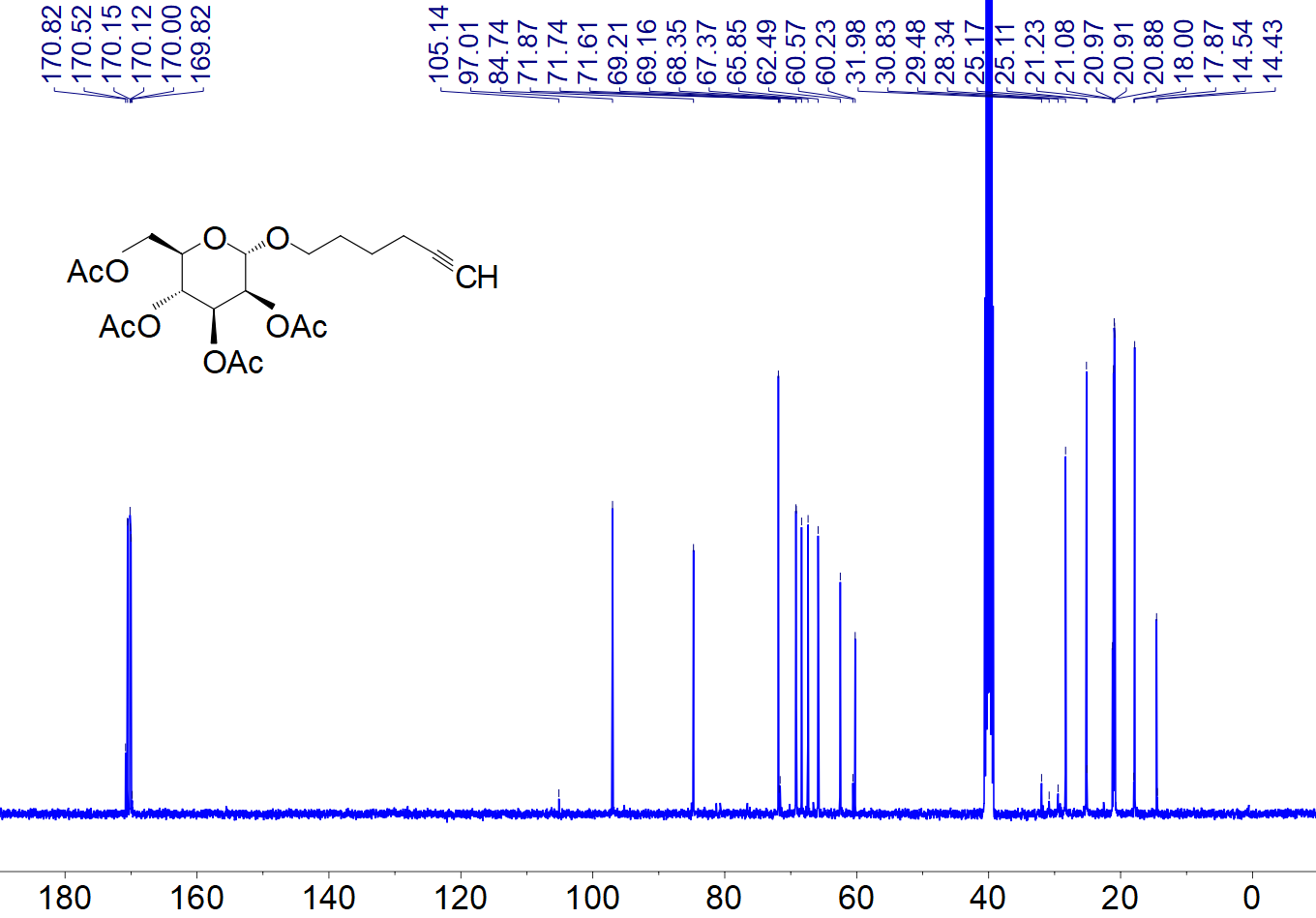


**Figure S2**. ^13^C NMR spectrum of **2b** in DMSO-*d*_6_*.*

**Figure S3.** ESI-MS spectrum of **2b**

NMR, Mass of 5-[1-(2,3,4,6-tetra-O-acetyl-α-D-Mannopyranoyl)-5-hexyn-6-yl]-2’-deoxy-5’-O-(4,4’-dimethoxytrityl)uridine (**3b**).


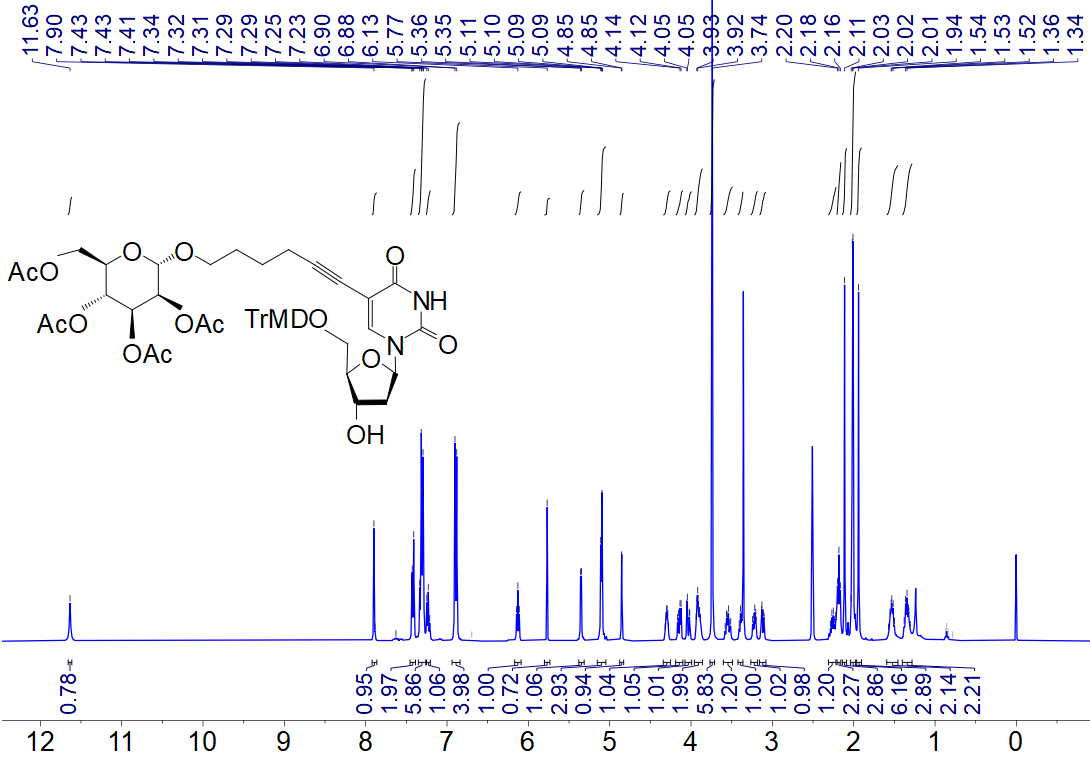


**Figure S4**. ^1^H NMR spectrum of **3b** in DMSO-*d6*


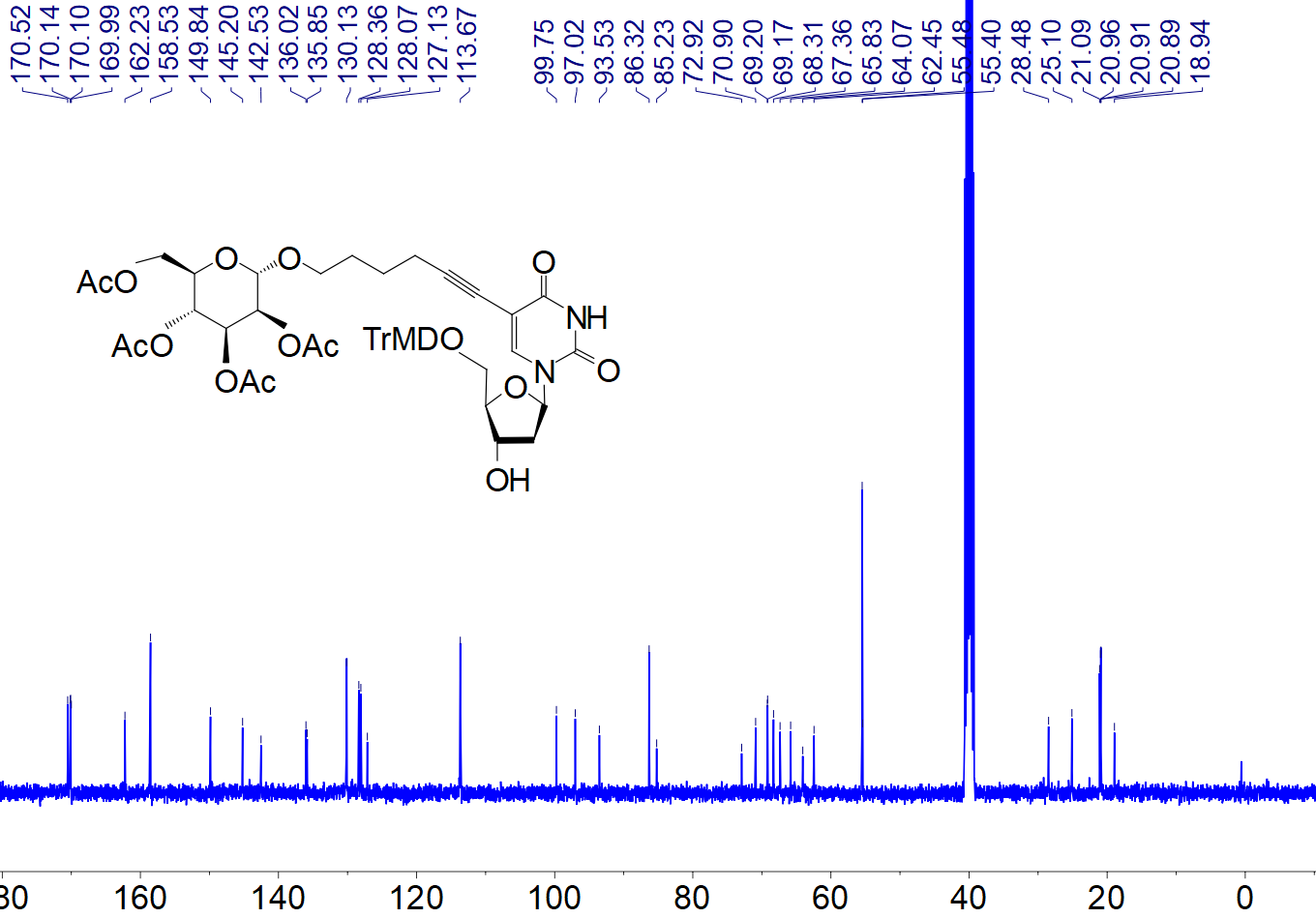


**Figure S5**. ^13^C NMR spectrum of **3b** in DMSO-*d6*

**Figure S6.** ESI-MS spectrum of **3b**

NMR, Mass of 5-(1-(2,3,4,6-tetra-O-acetyl-α-D-Mannopyranoyl)-5-hexyn-6-yl)-2’-deoxy-5’-O-(4,4’-dimethoxytrityl)uridine-3’-O-(2-cyanoethyl-N,N’-diisopropyl)-phosphoramidite (**4b**).


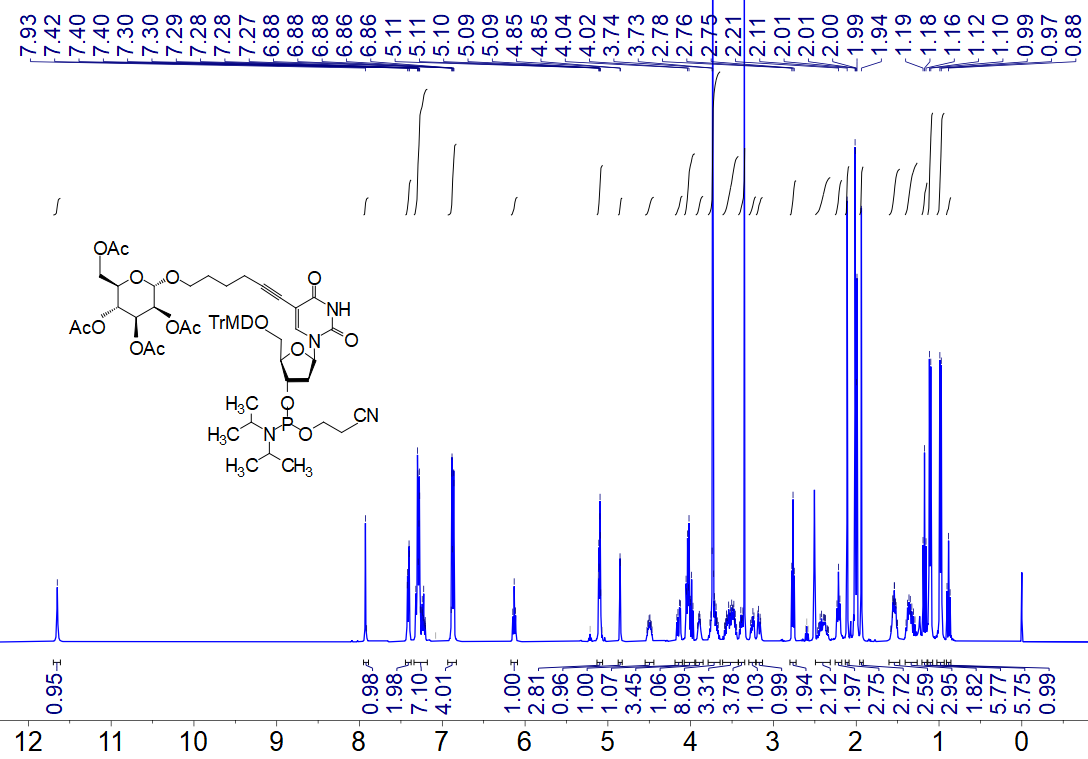


**Figure S7**. ^1^H NMR spectrum of **4b** in DMSO-*d_6_*


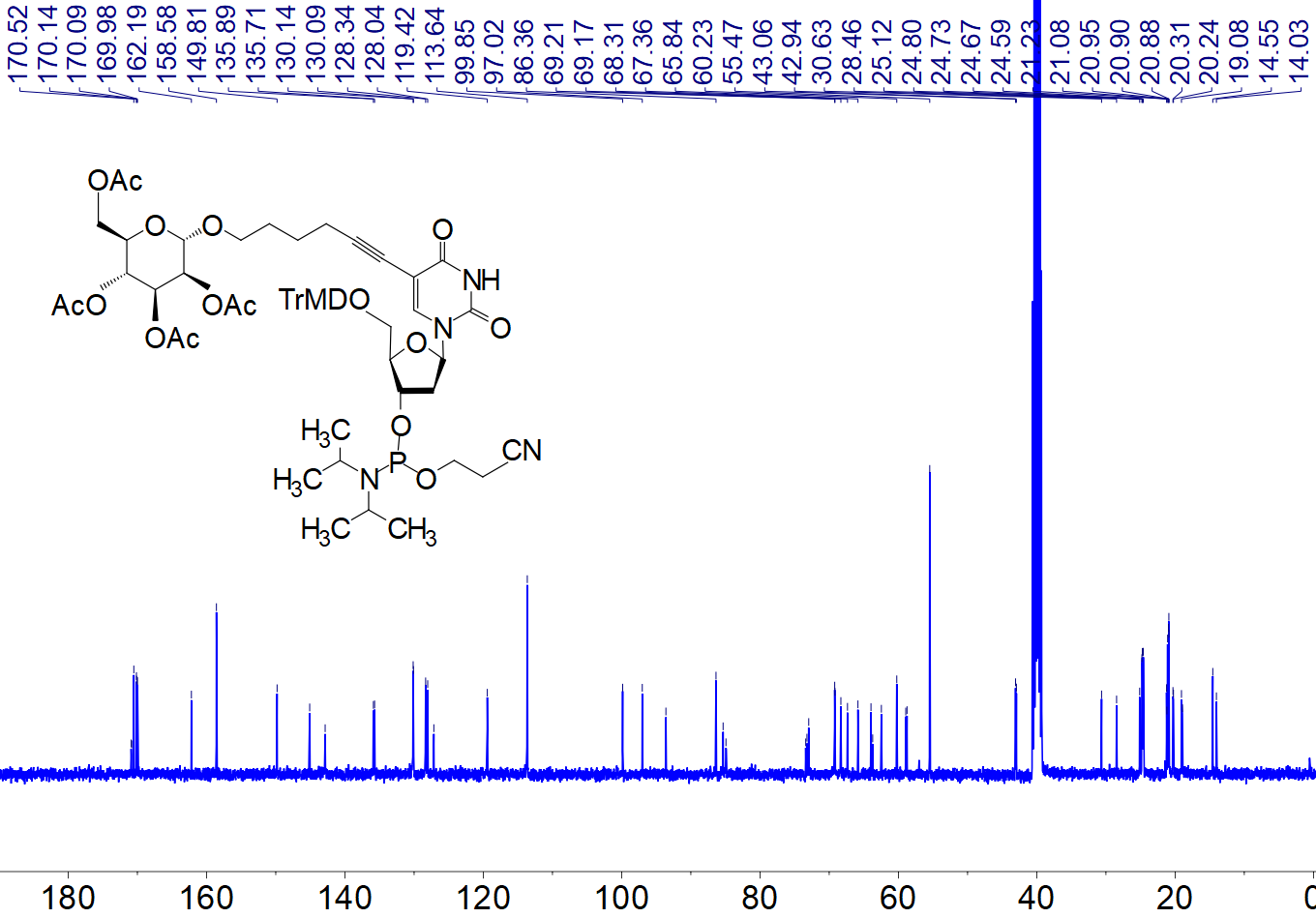


**Figure S8**. ^13^C NMR spectrum of **4b** in DMSO-*d_6_*


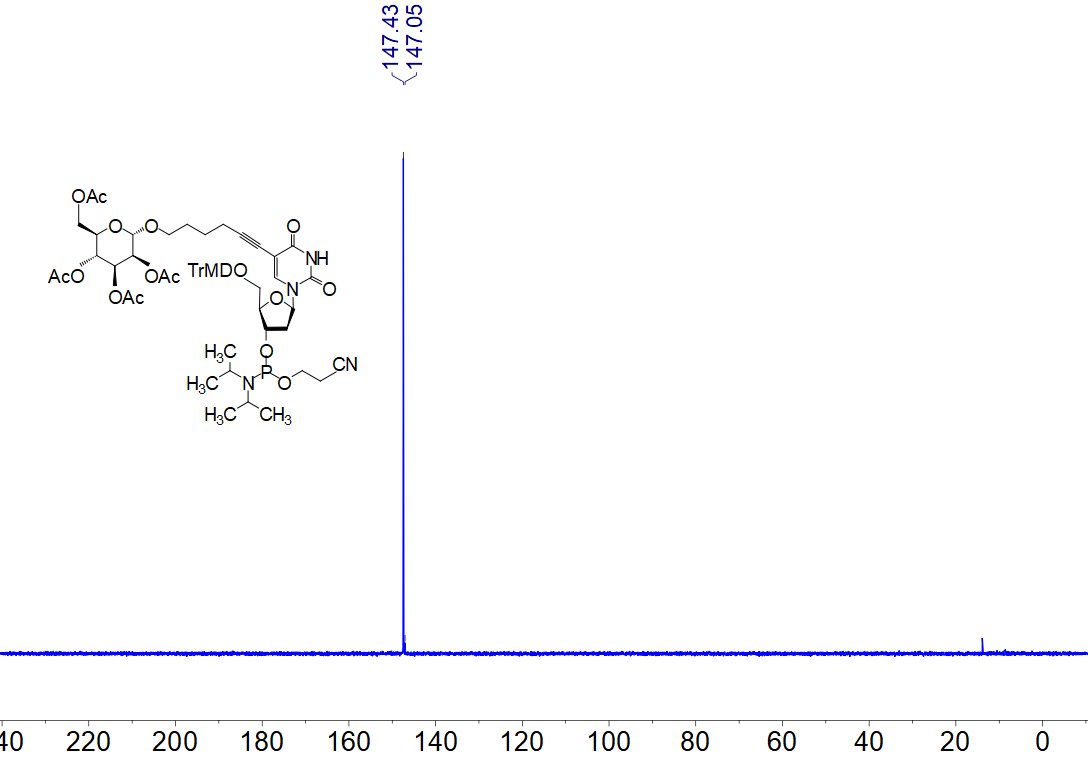


**Figure S9**. ^31^P NMR spectrum of **4b** in DMSO-*d_6_*

**Figure S10.** ESI-MS spectrum of **4b**

NMR and Mass of 1-(5-Hexyn-1-yl)-2,3,4,6-tetra-O-acetyl-β-D-Glucopyranoside (**2c**).


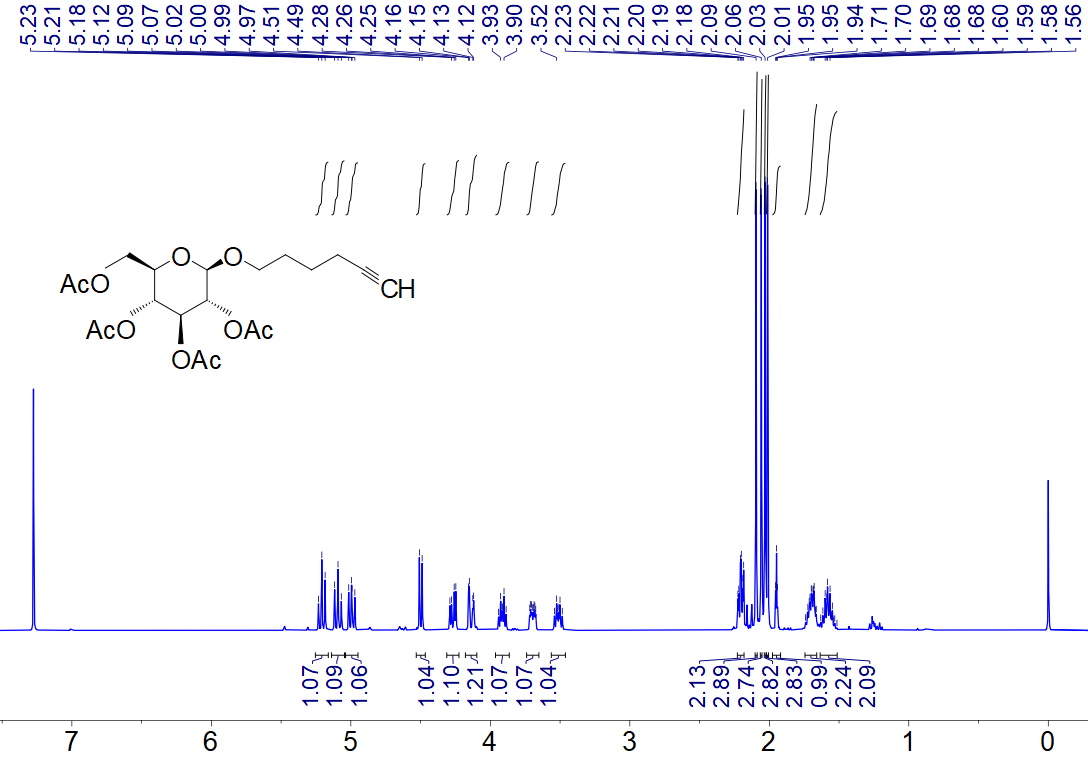


**Figure S11**. ^1^H NMR spectrum of **2c** in Chloroform-*d.*


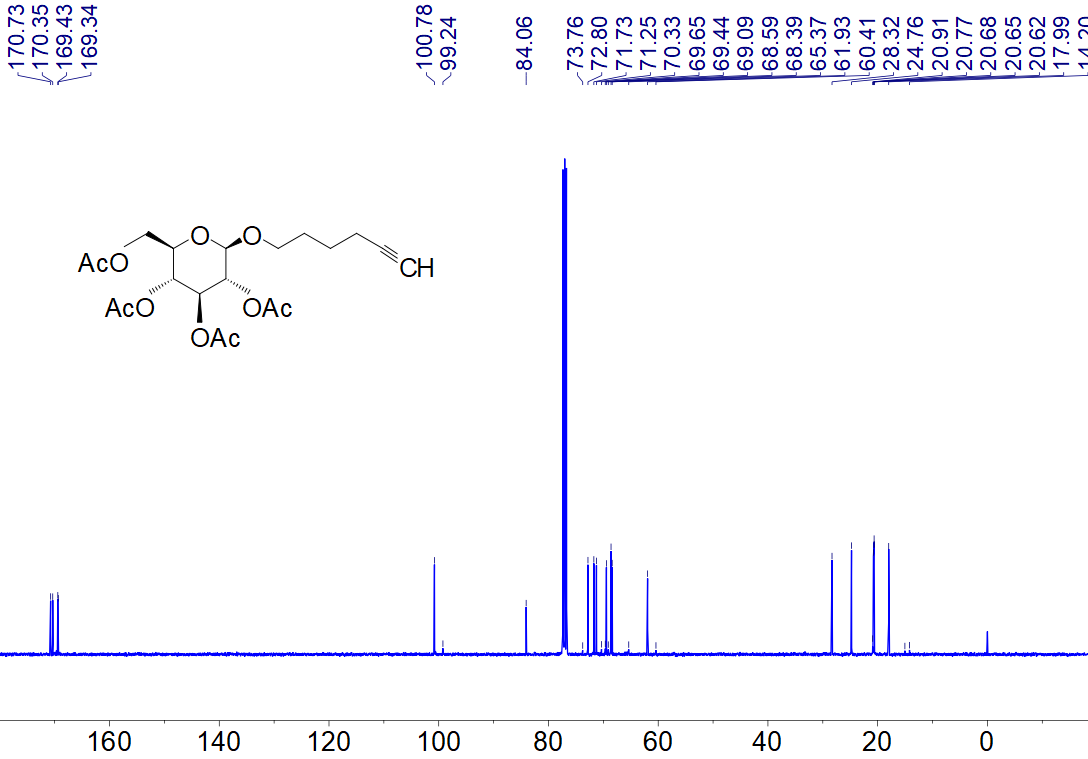


**Figure S12**. ^13^C NMR spectrum of **2c** in Chloroform-*d.*

**Figure S13.** ESI-MS spectrum of **2c**

NMR and Mass of 5-[1-(2,3,4,6-tetra-O-acetyl-β-D-Glucopyranoyl)-5-hexyn-6-yl]-2’-deoxy-5’-O-(4,4’-dimethoxytrityl)uridine (**3c**).

*
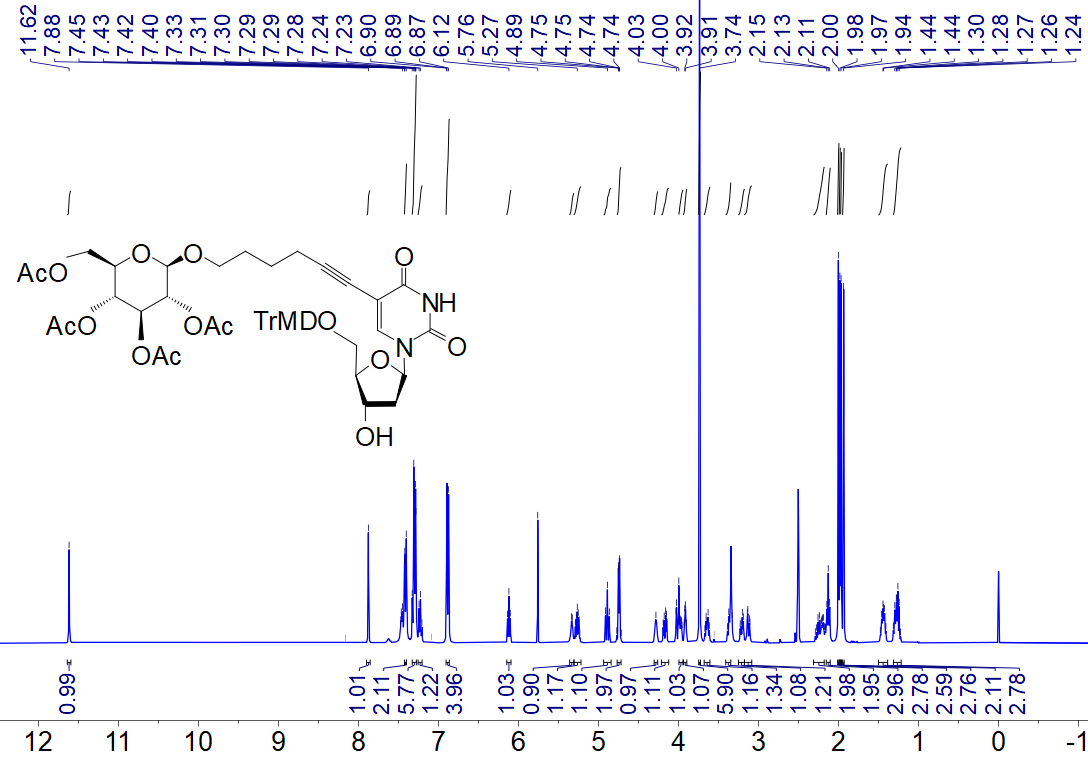
*

**Figure S14**. ^1^H NMR spectrum of **3c** in DMSO-*d_6_*


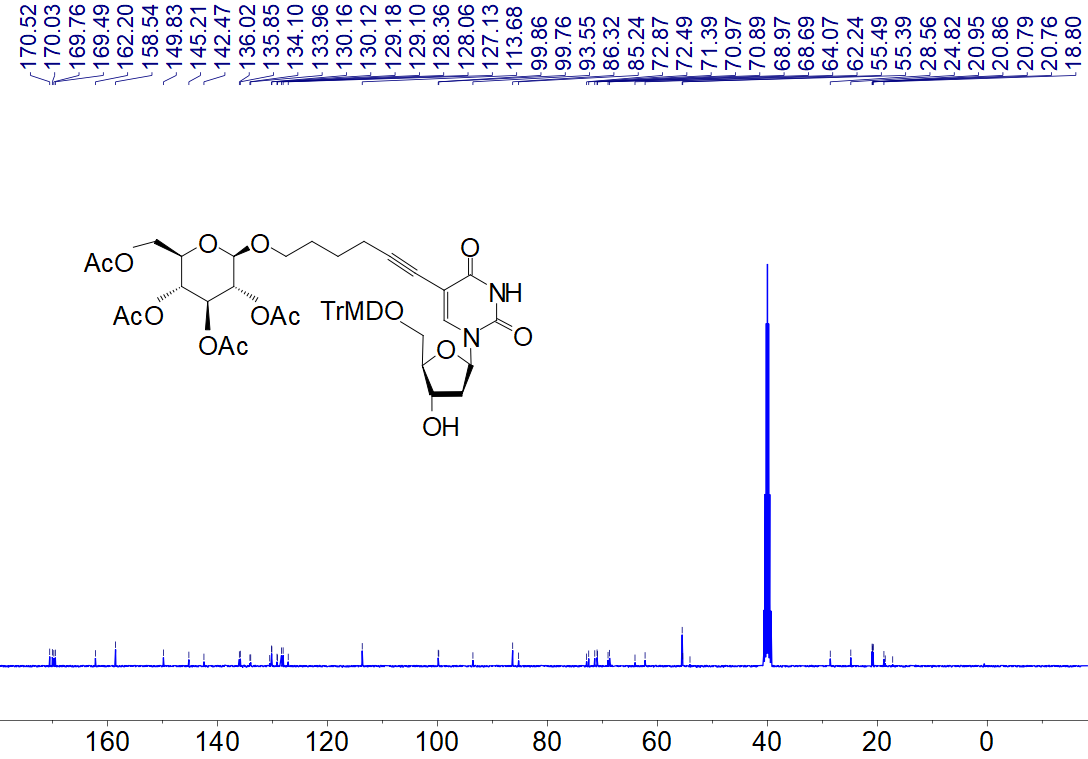


**Figure S15**. ^13^C NMR spectrum of **3c** in DMSO-*d_6_*

**Figure S16.** ESI-MS spectrum of **3c**

NMR and Mass of 5-(1-(2,3,4,6-tetra-O-acetyl-β-D-Glucopyranoyl)-5-hexyn-6-yl)-2’-deoxy-5’-O-(4,4’-dimethoxytrityl)uridine-3’-O-(2-cyanoethyl-N,N’-diisopropyl)-phosphoramidite **(4c)**


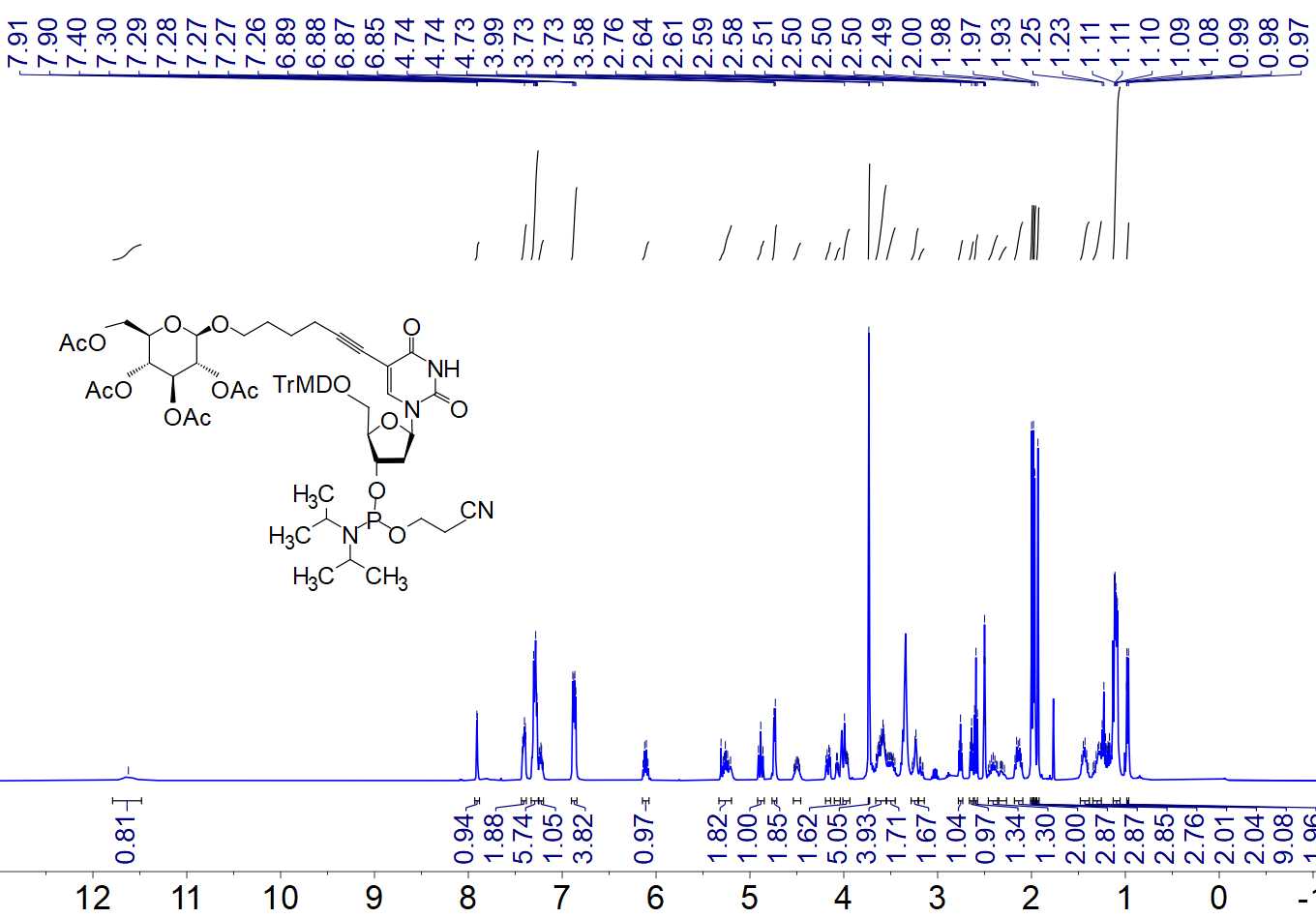


**Figure S17**. ^1^H NMR spectrum of **4c** in DMSO-*d_6_*


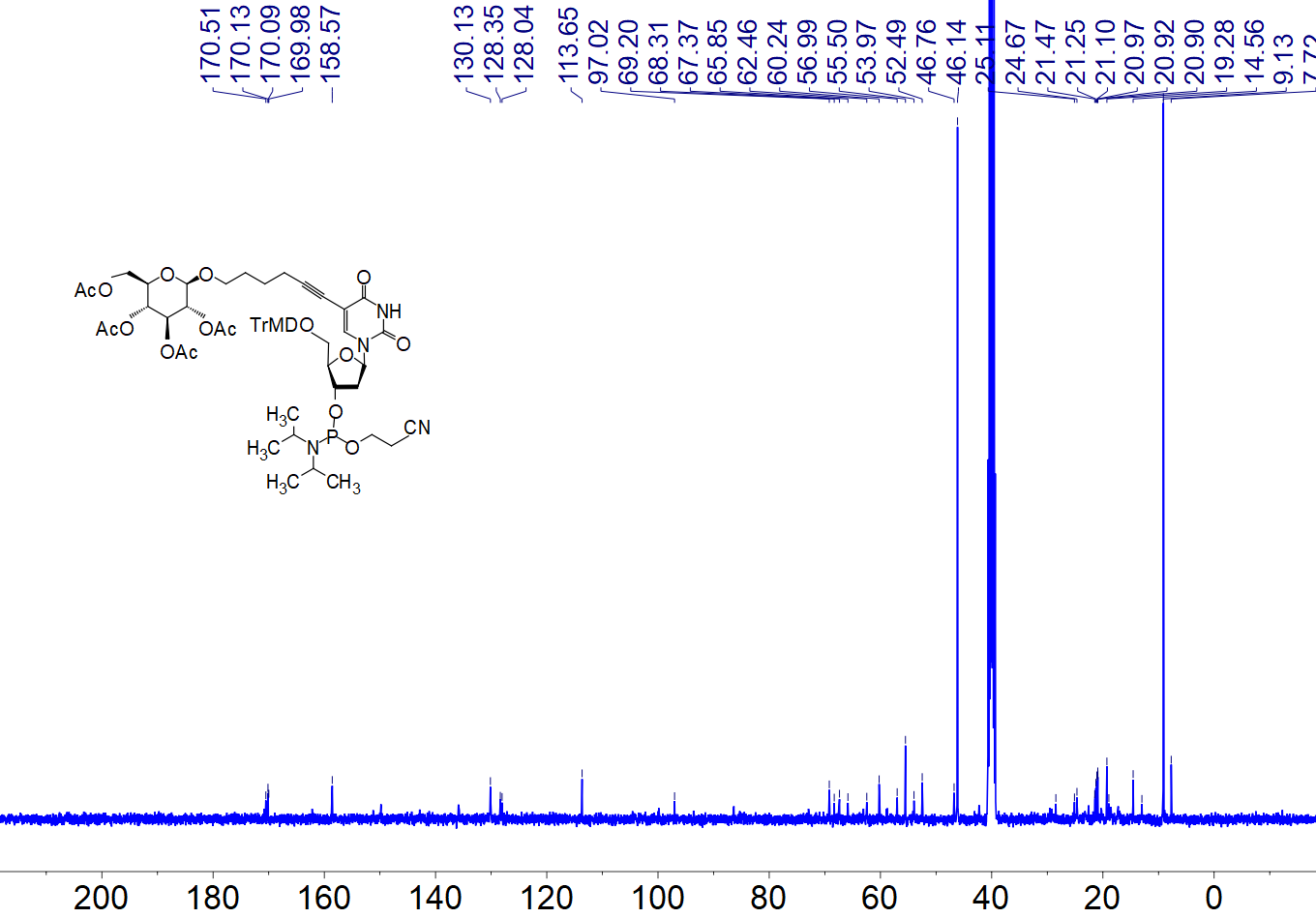


**Figure S18**. ^13^C NMR spectrum of **4c** in DMSO-*d_6_*


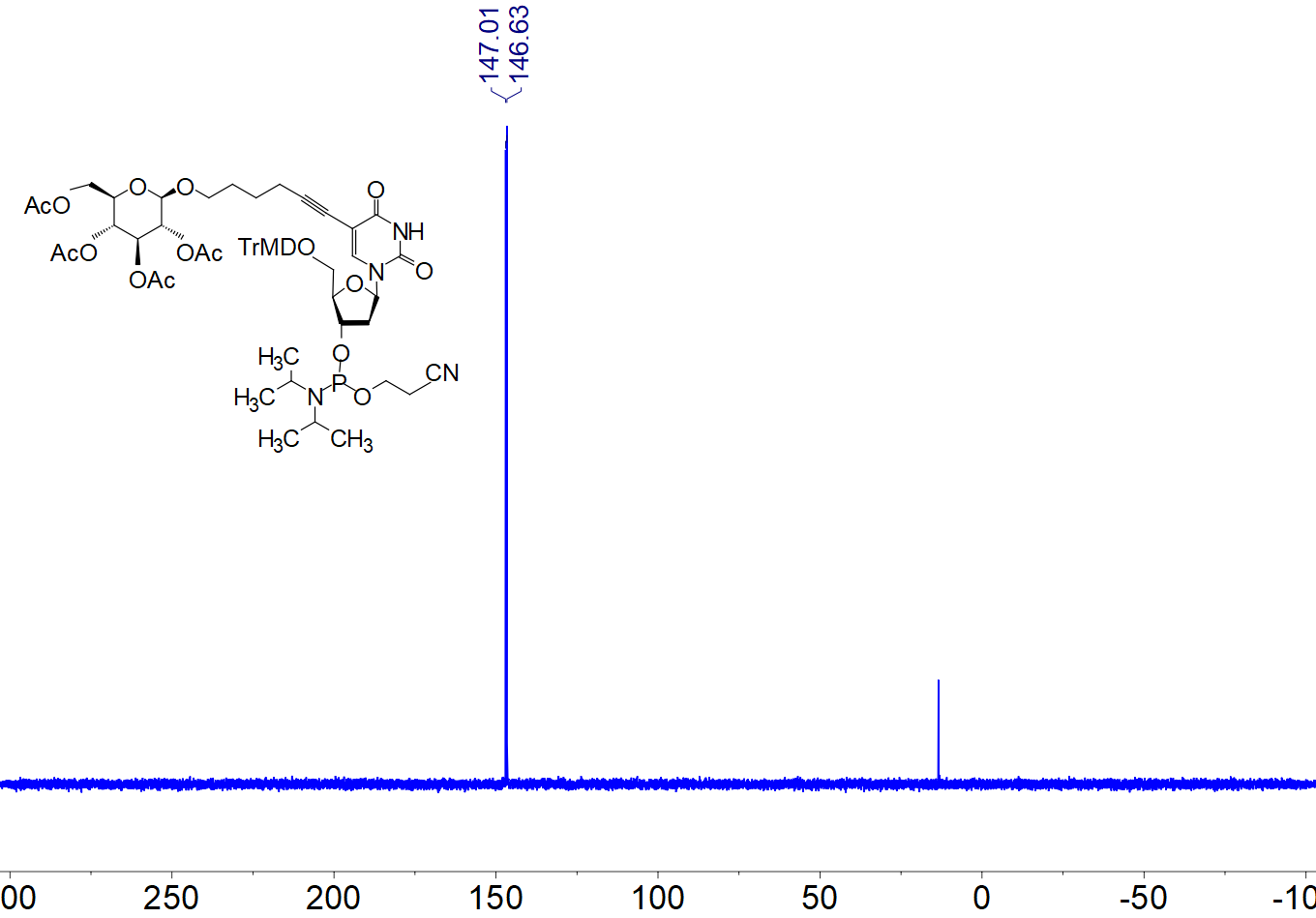


**Figure S19**. ^31^P NMR spectrum of **4c** in DMSO-*d_6_*

**Figure S20.** ESI-MS spectrum of **4c**

NMR and Mass of 1-(5-Hexyn-1-yl)-2-Acetamido-3,4,6-tri-O-acetyl-β-D-Galactopyranoside **(2d).**


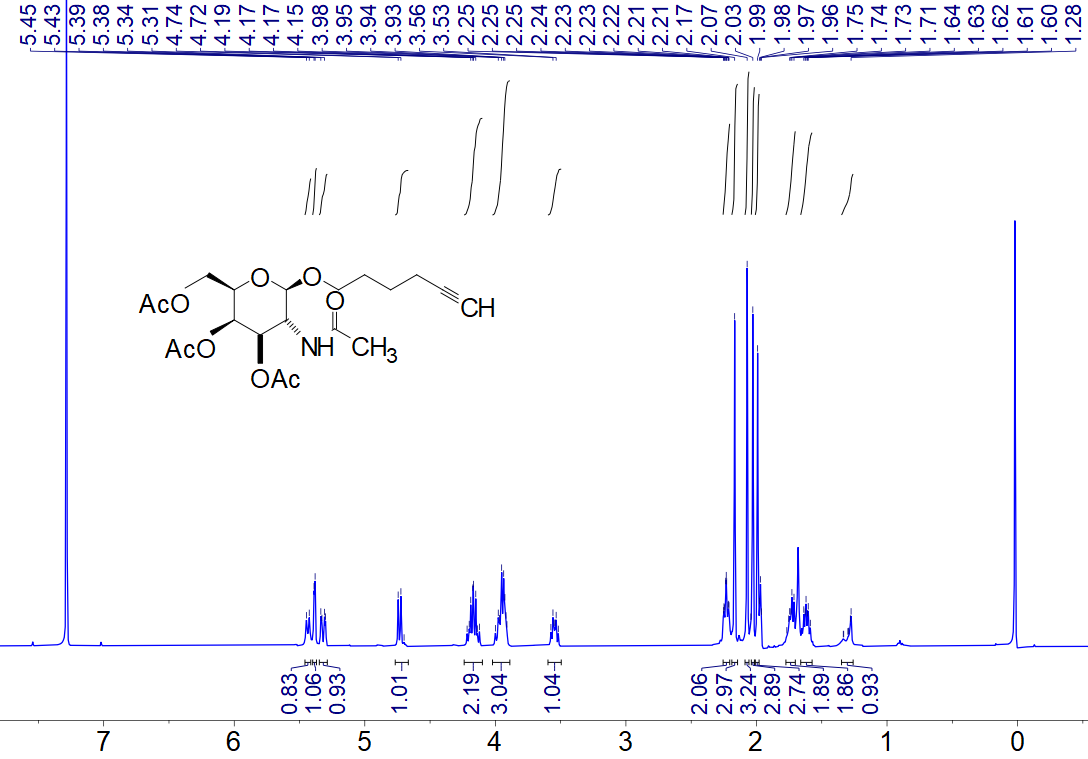


**Figure S21**. ^1^H NMR spectrum of **2d** in Chloroform-*d.*


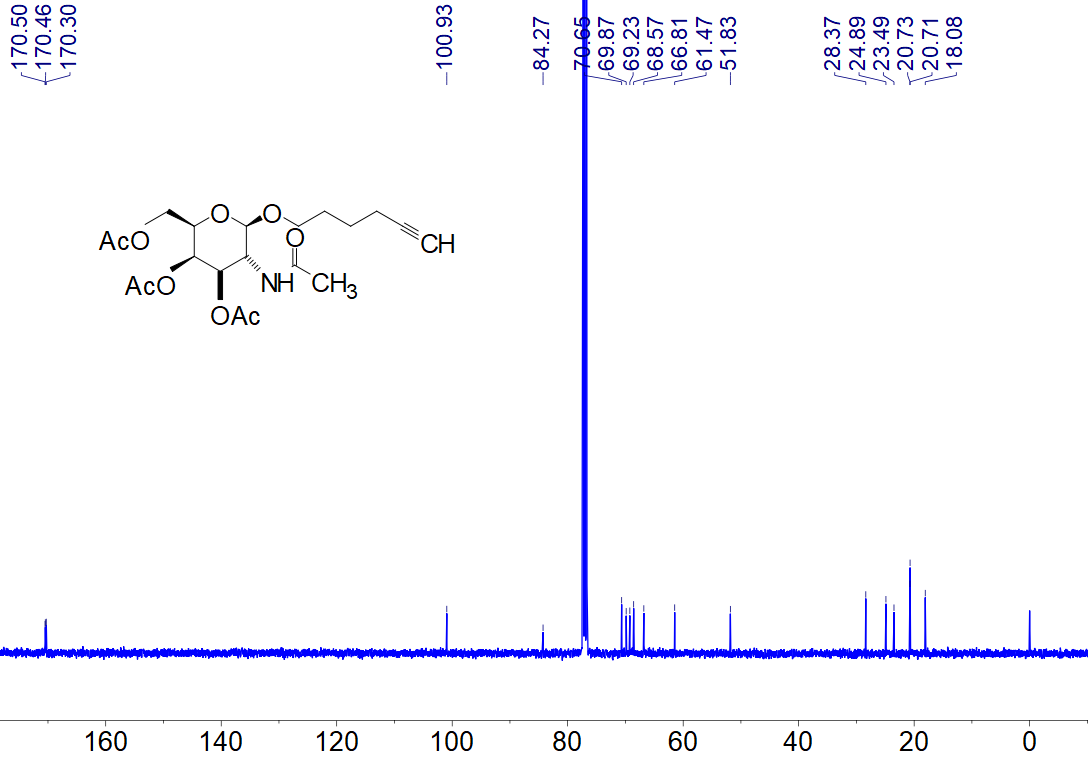


**Figure S22**. ^13^C NMR spectrum of **2d** in Chloroform-*d.*

**Figure S23.** ESI-MS spectrum of **2d**

NMR and Mass of 5-[1-(2-Acetamido-3,4,6-tri-O-acetyl-β-D-Galactopyranosyl)-5-hexyn-6-yl]-2’-deoxy-5’-O-(4,4’-dimethoxytrityl)uridine (**3d**)**.**


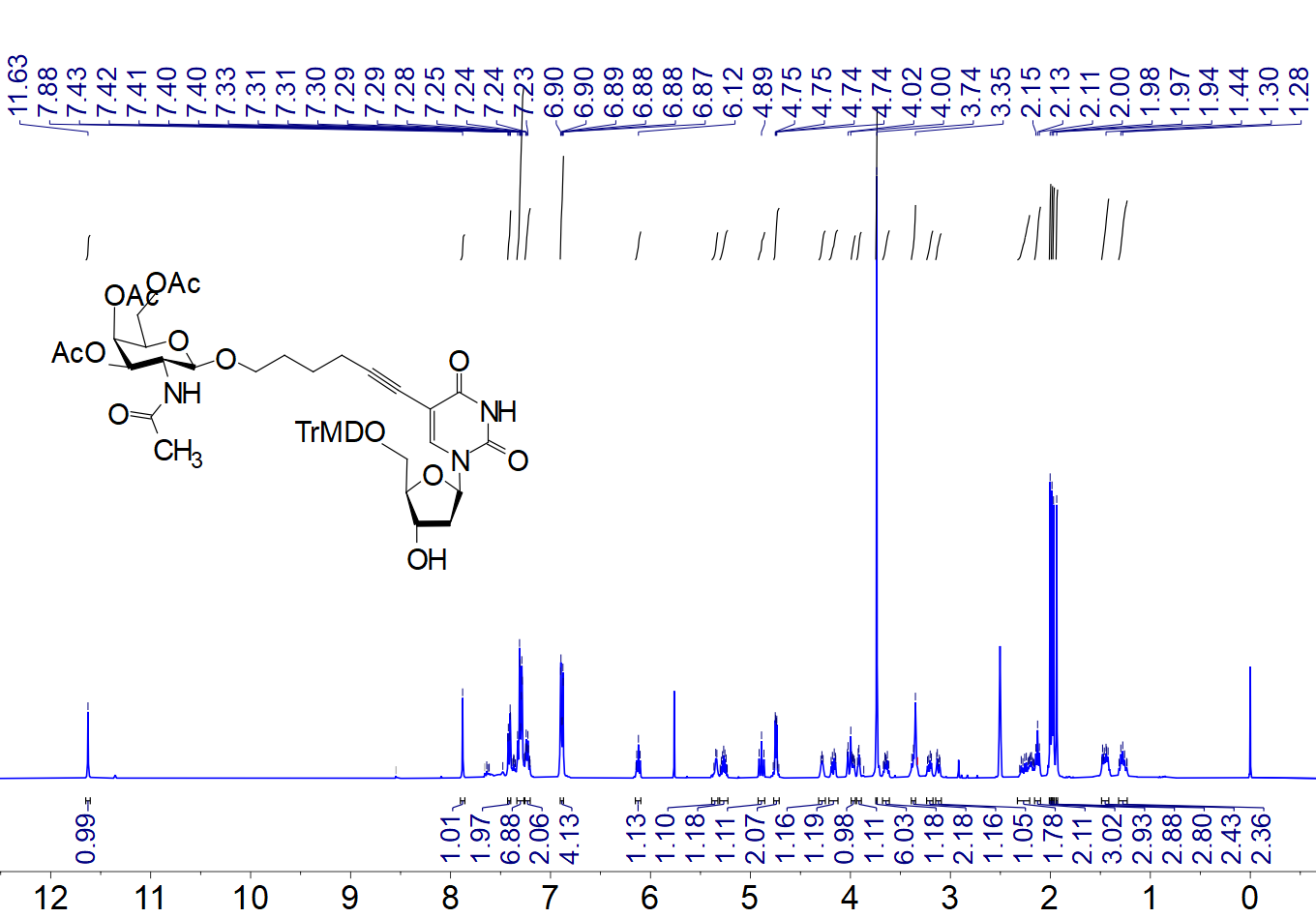


**Figure S24**. ^1^H NMR spectrum of **3d** in DMSO-*d_6_*


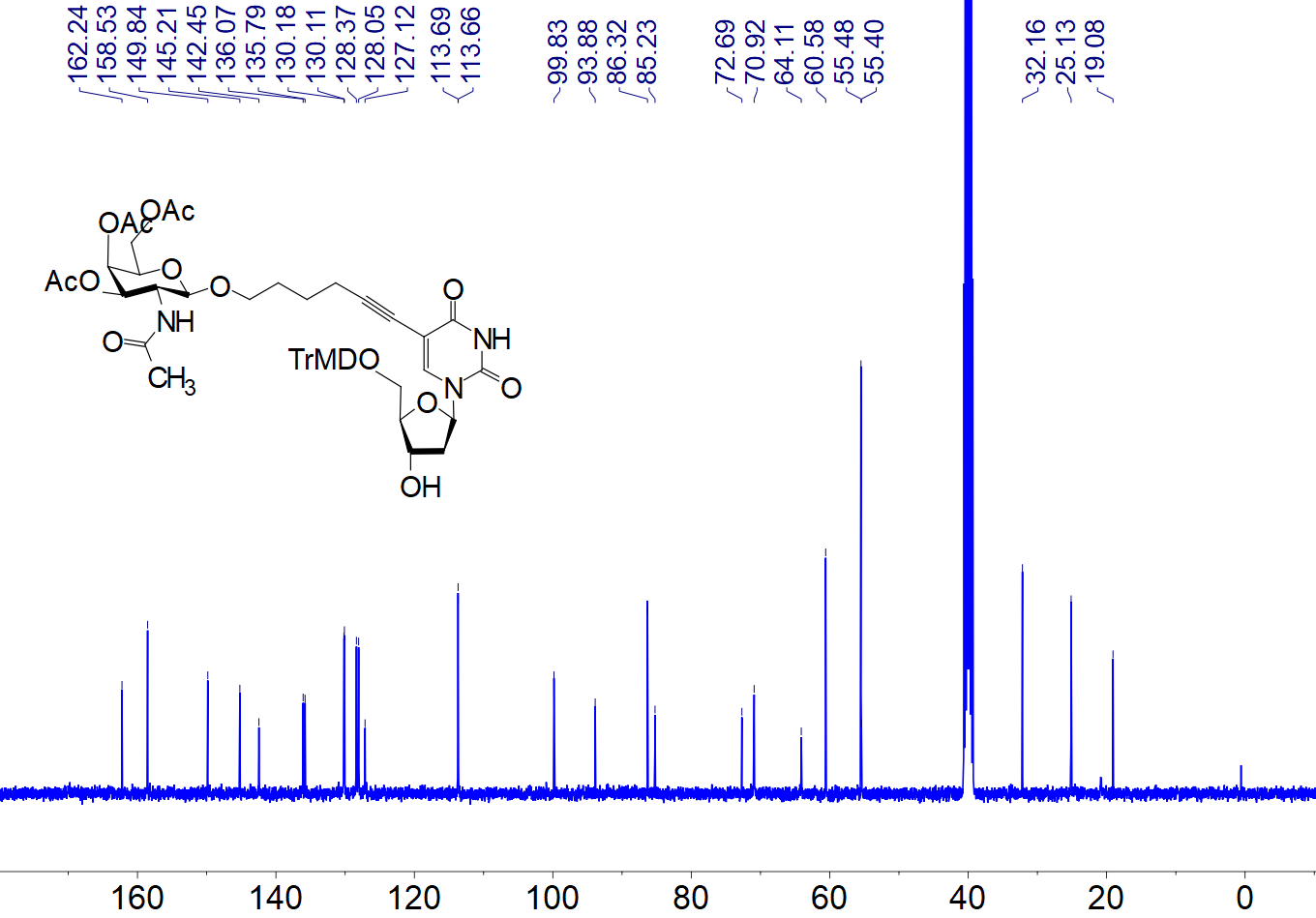


**Figure S25**. ^13^C NMR spectrum of **3d** in DMSO-*d_6_*

**Figure S26.** ESI-MS spectrum of **3d**

NMR and Mass of 5-(1-(2-Acetamido-3,4,6-tri-O-acetyl-β-D-Galactopyranosyl)-5-hexyn-6-yl)-2’-deoxy-5’-O-(4,4’-dimethoxytrityl)uridine-3’-O-(2-cyanoethyl-N,N’-diisopropyl)-phosphoramidite (**4d**).


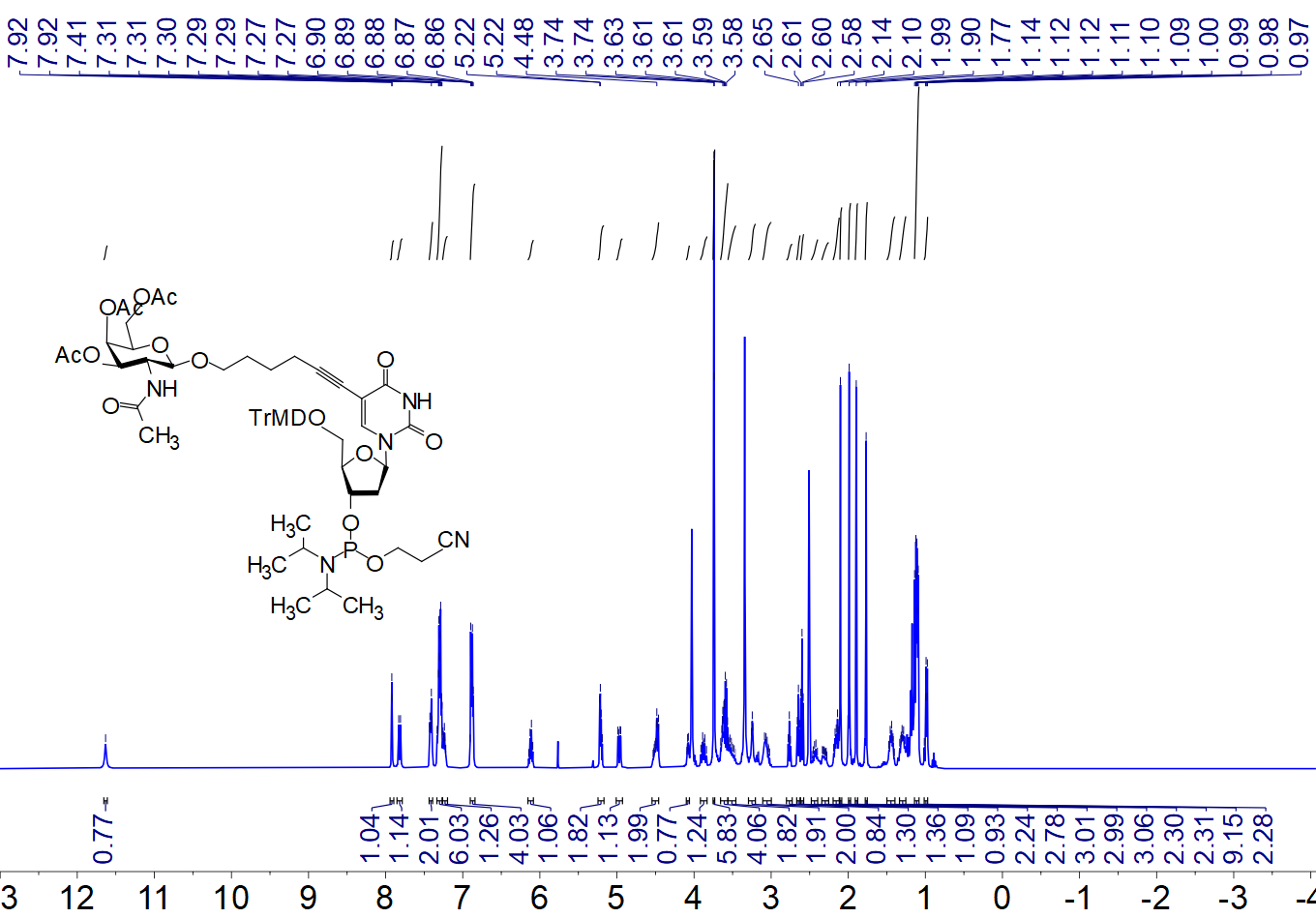


**Figure S27**. ^1^H NMR spectrum of **4d** in DMSO-*d_6_*


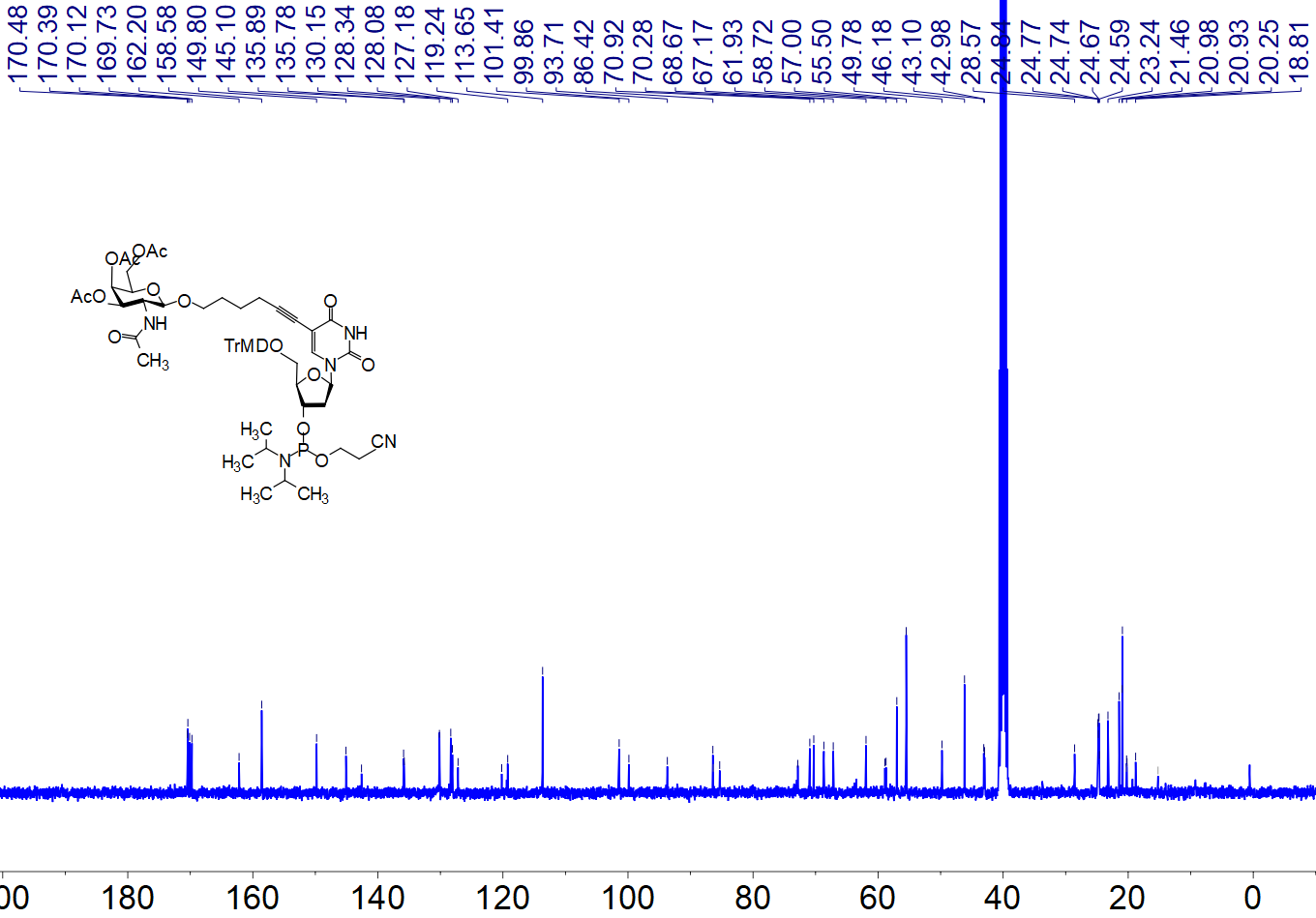


**Figure S28**. ^13^C NMR spectrum of **4d** in DMSO-*d_6_*


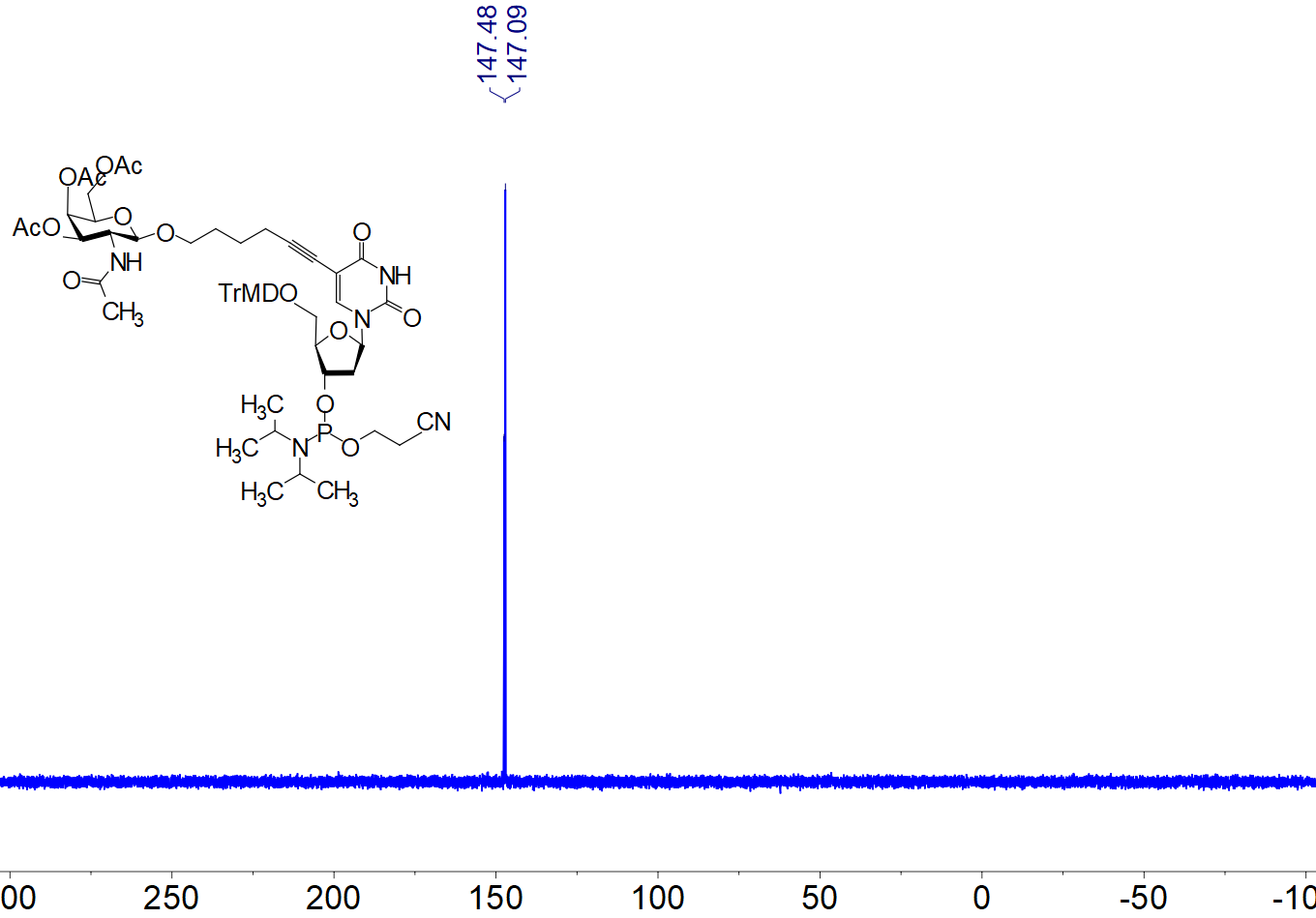


**Figure S29**. ^31^P NMR spectrum of **4d** in DMSO-*d_6_*

**Figure S30.** ESI-MS spectrum of **4d**

**Mass of GNAAs**


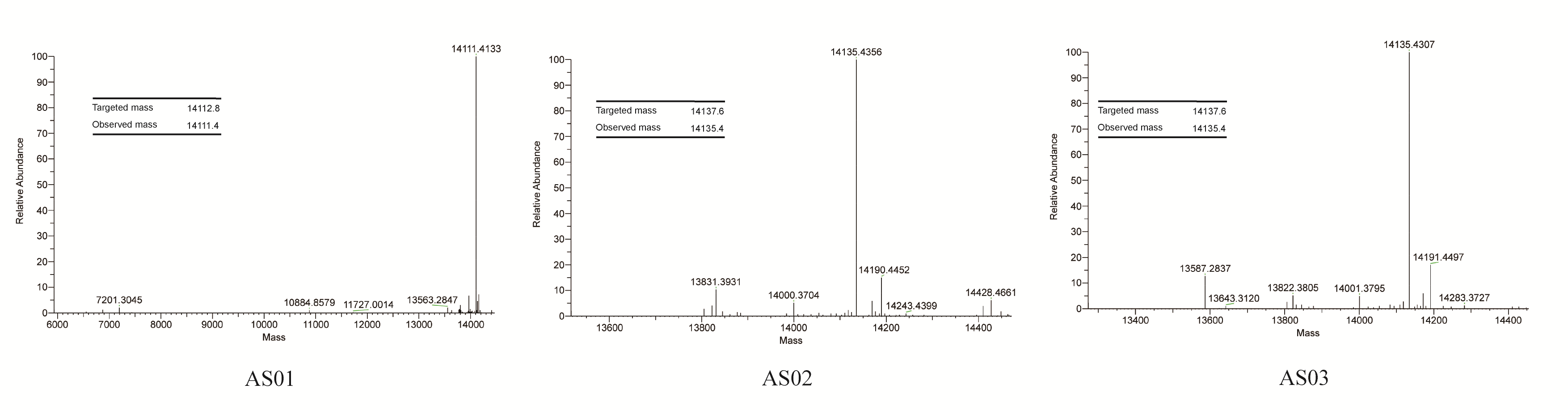


**Figure S31.** Mass of Gal-Sgc8s.


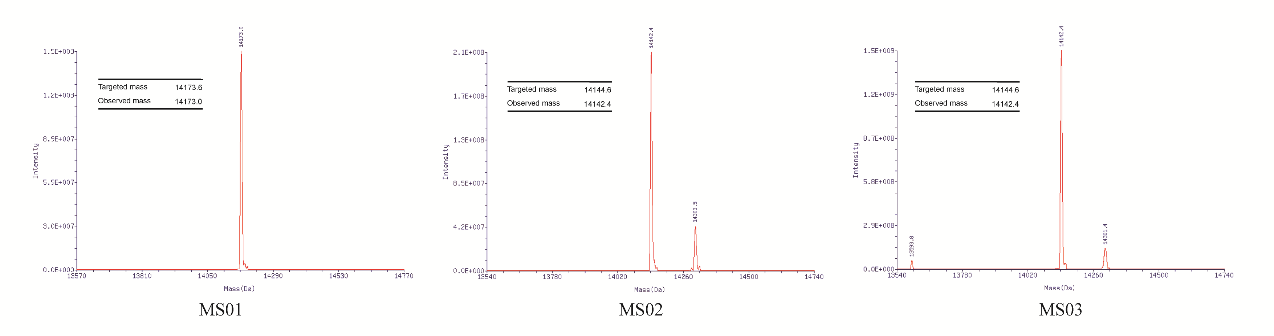


**Figure S32.** Mass of Man-Sgc8s.


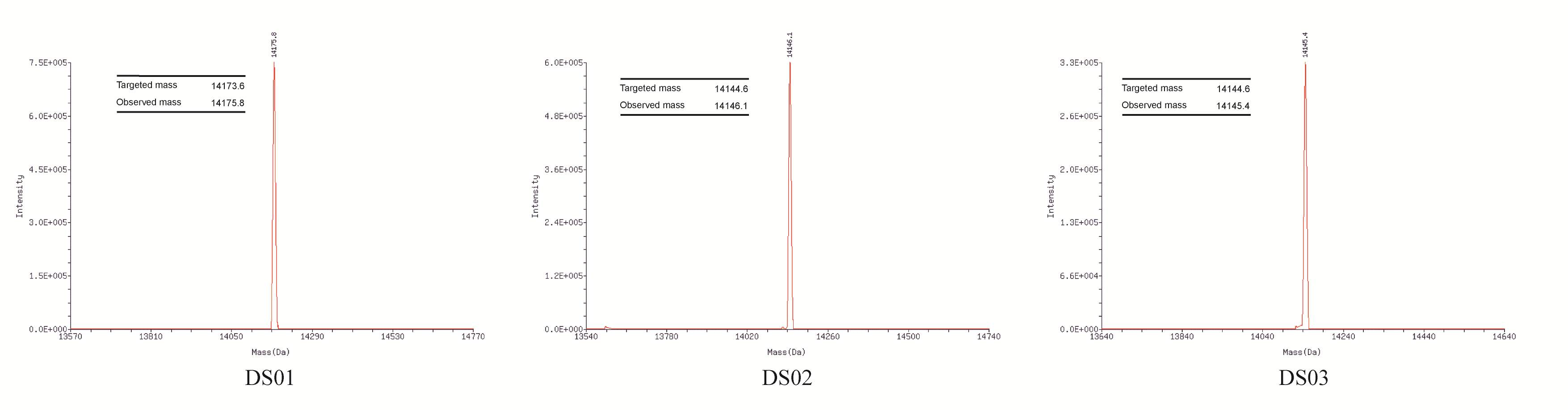


**Figure S33.** Mass of Glu-Sgc8s.


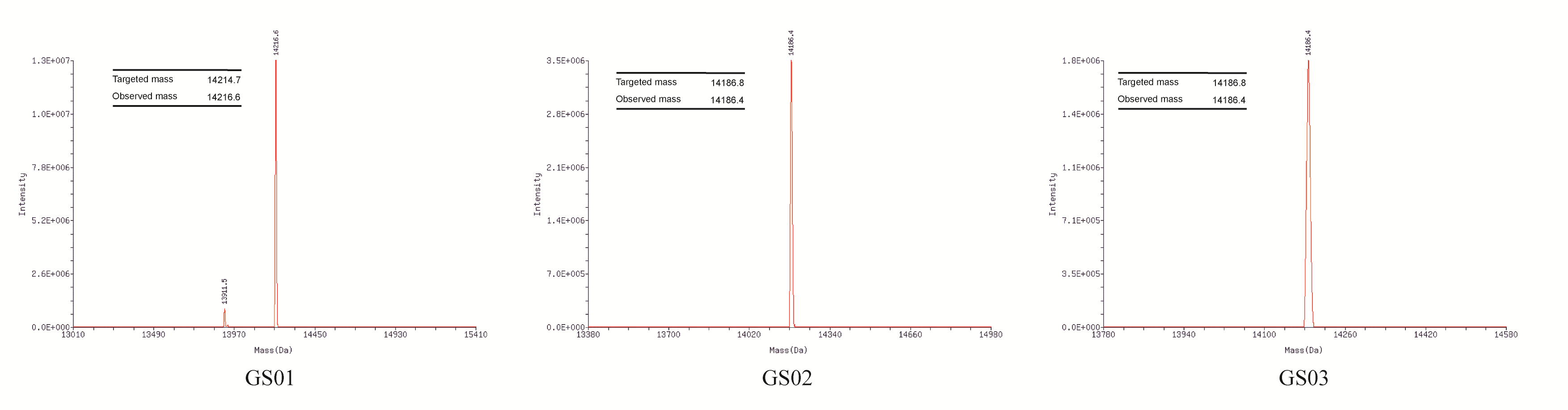


**Figure S34.** Mass of GalN-Sgc8s.


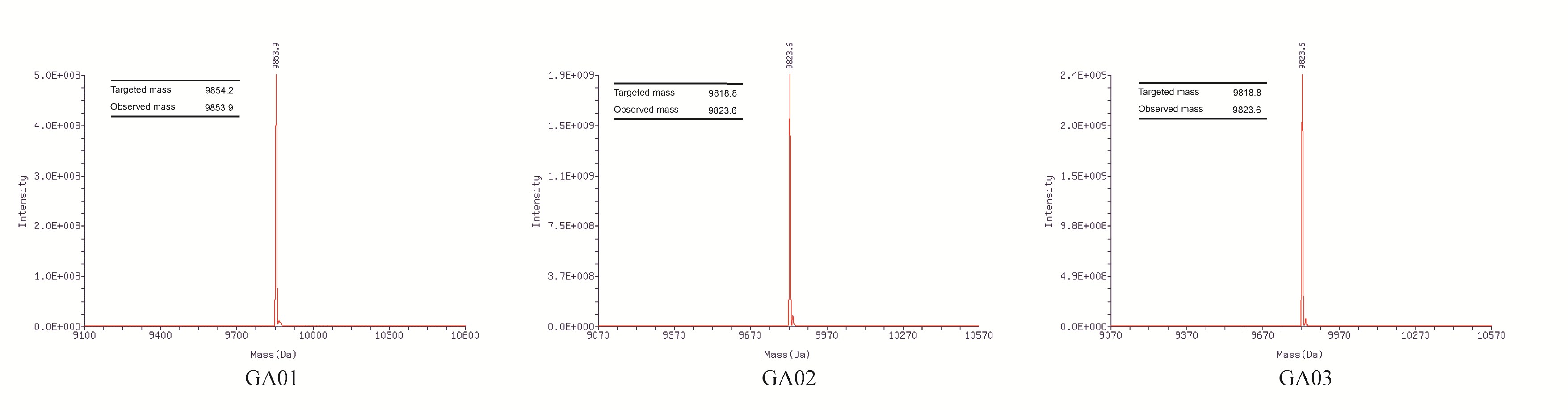


**Figure S35.** Mass of GalN-AS1411s.


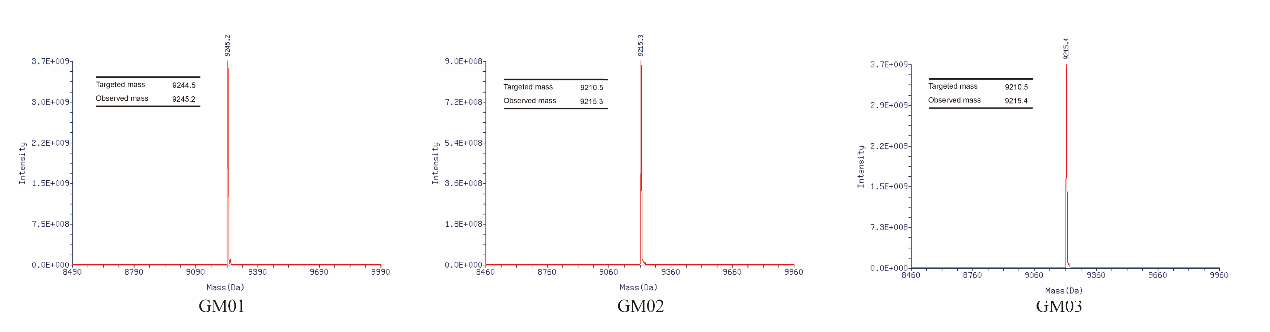


**Figure S36.** Mass of GalN-MUC1s.


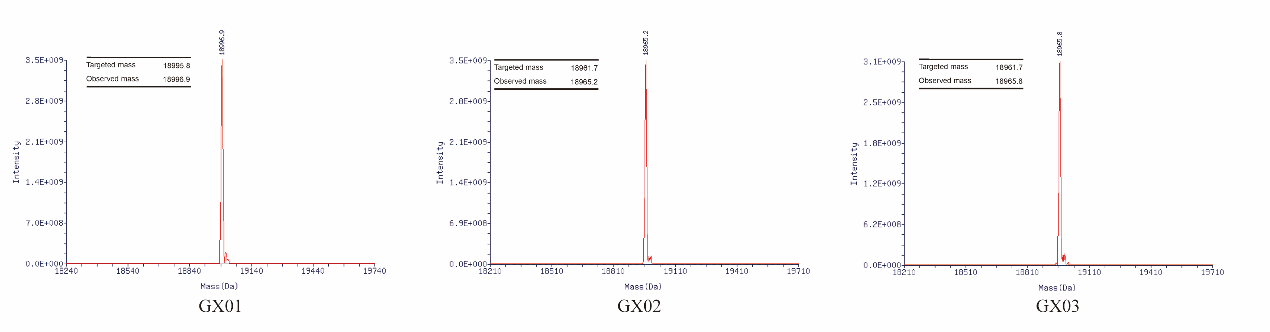


**Figure S37.** Mass of Gal-XQ-2Ds.

**Figure S38.** Stability of GNAAs and invert dT modified aptamer after incubation with 10% FBS for 72 h analysis by PAGE and fluorescence quantitative analysis. GS03: 3’ GalNAC modification; IS01: 3’ invert dT modification;


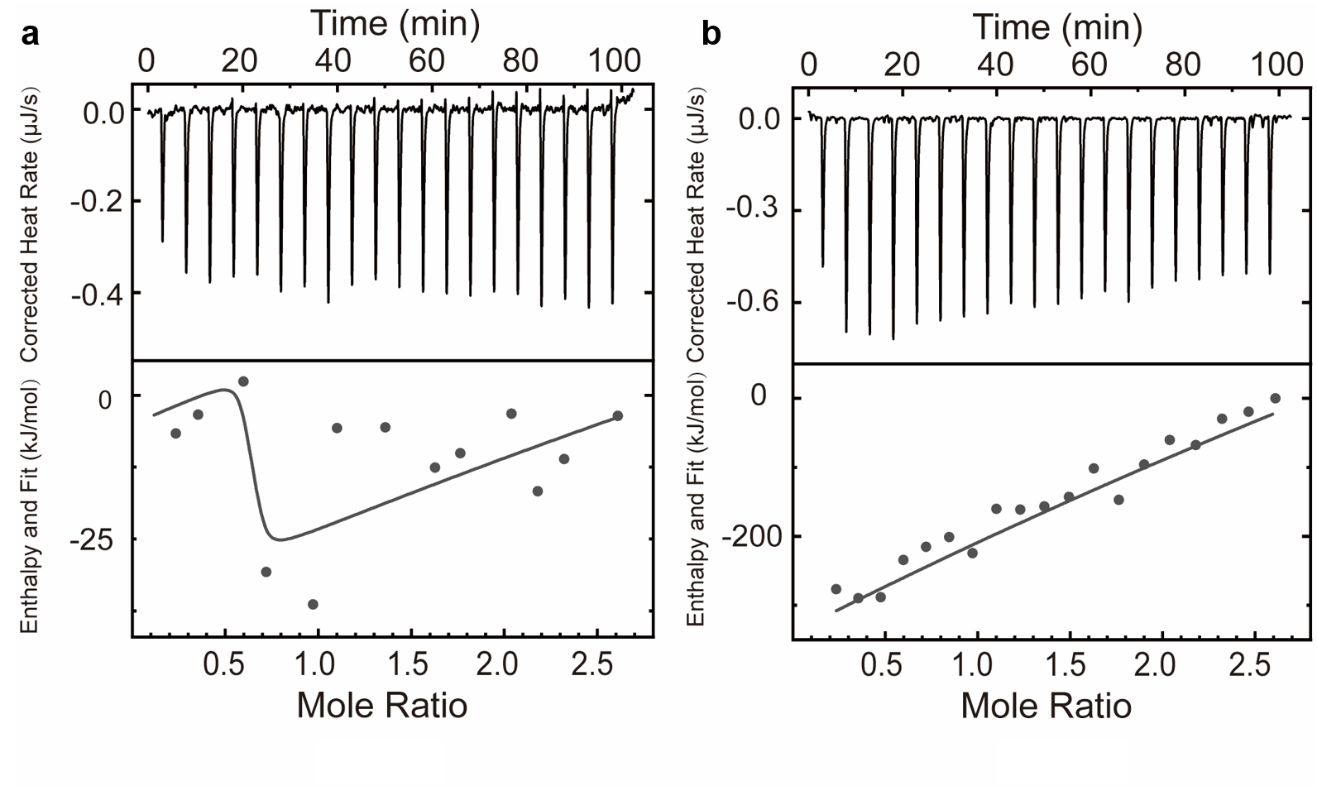


**Figure S39.** Isothermal titration calorimetric curves of (a) S01 and (b) GS01 with Exo1.


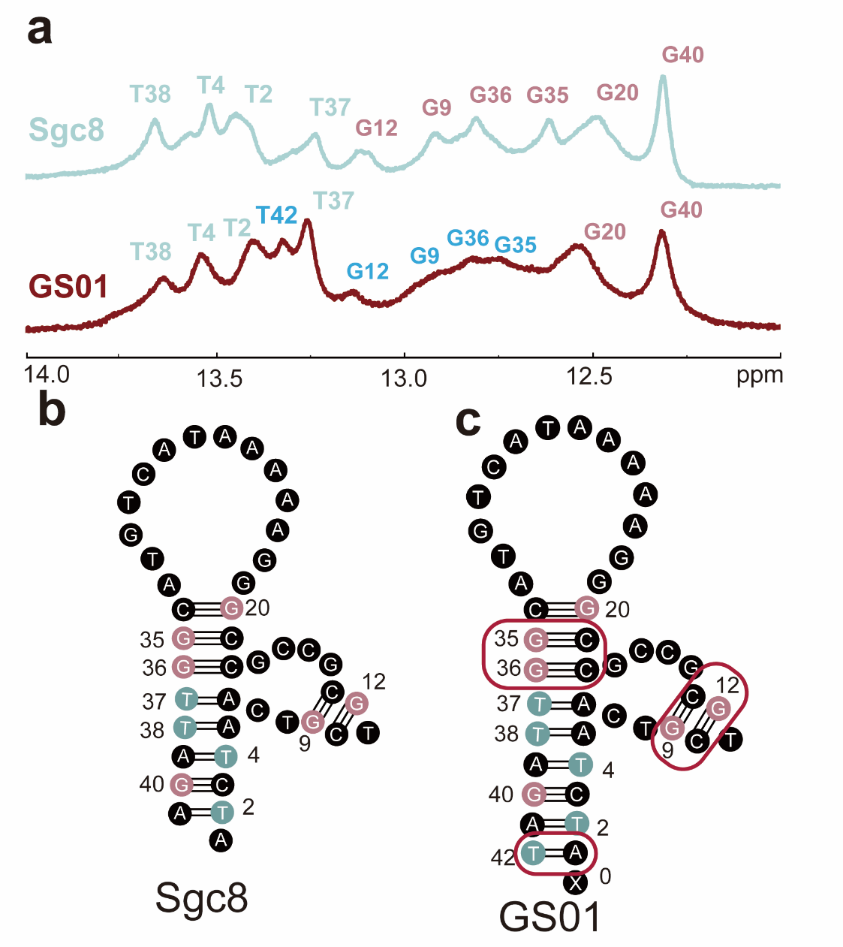


**Figure S40.** (a) The ^1^H NMR spectra involve G H1 and T H3 signals of Sgc8 and GS01. [DNA] = 0.06 mM, [NaPi, pH 7] = 10 mM, [MgCl_2_] = 5 mM, 90% H_2_O/10% D_2_O, T = 4 °C. (b) Schematic for the simplified secondary structure of Sgc8. (c) Schematic for the simplified secondary structure of GS01, with red rectangles marking the sites that differ from Sgc8.


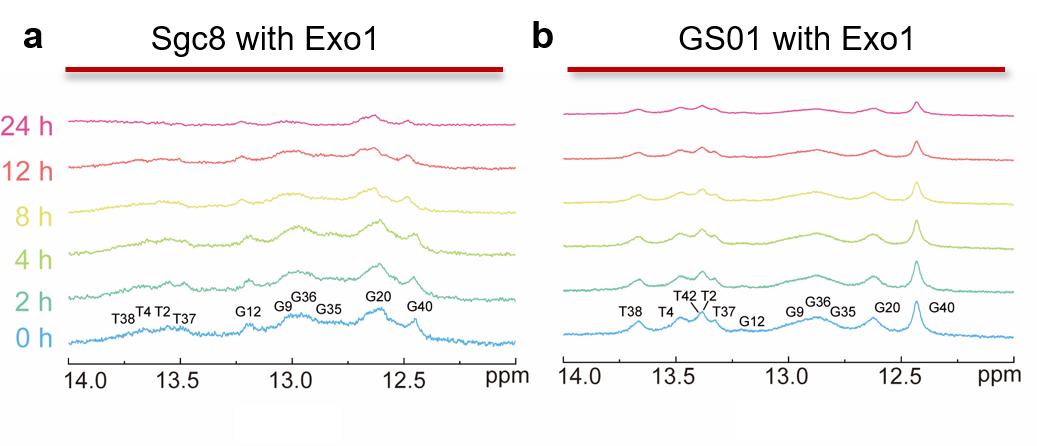


**Figure S41.** The 1H NMR spectra of (a) S01 and (b) GS01 after incubation with Exo1 (10 μM) for different time.

**Figure S42.** The effects of GNAAs on the activity of HEK293T were determined by CCK-8.
